# Optogenetic induction of TDP-43 aggregation impairs neuronal integrity and behavior in *Caenorhabditis elegans*

**DOI:** 10.1186/s40035-025-00480-x

**Published:** 2025-04-16

**Authors:** Kyung Hwan Park, Euihyeon Yu, Sooji Choi, Sangyeong Kim, Chanbin Park, J. Eugene Lee, Kyung Won Kim

**Affiliations:** 1 Department of Life Science, Multidisciplinary Genome Institute, Hallym University, Chuncheon, South Korea; 2 Biometrology Group, Division of Biomedical Metrology, Korea Research Institute of Standards and Science, Daejeon, South Korea; 3 Graduate School of Analytical Science and Technology, Chungnam National University, Daejeon, South Korea

**Keywords:** Neurodegenerative diseases, Optogenetics, OptoDroplet, TDP-43 proteinopathy, Amyotrophic lateral sclerosis, Frontotemporal lobar degeneration, *C. elegans*

## Abstract

**Background:**

Cytoplasmic aggregation of TAR DNA binding protein 43 (TDP-43) in neurons is one of the hallmarks of TDP-43 proteinopathy. Amyotrophic lateral sclerosis (ALS) and frontotemporal lobar degeneration (FTLD) are closely associated with TDP-43 proteinopathy; however, it remains uncertain whether TDP-43 aggregation initiates the pathology or is a consequence of it.

**Methods:**

To demonstrate the pathology of TDP-43 aggregation, we applied the optoDroplet technique in *Caenorhabditis elegans* (*C. elegans*), which allows spatiotemporal modulation of TDP-43 phase separation and assembly.

**Results:**

We demonstrate that optogenetically induced TDP-43 aggregates exhibited insolubility similar to that observed in TDP-43 proteinopathy. These aggregates increased the severity of neurodegeneration, particularly in GABAergic motor neurons, and exacerbated sensorimotor dysfunction in *C. elegans*.

**Conclusions:**

We present an optogenetic *C. elegans* model of TDP-43 proteinopathy that provides insight into the neuropathological mechanisms of TDP-43 aggregates. Our model serves as a promising tool for identifying therapeutic targets for TDP-43 proteinopathy.

**Supplementary Information:**

The online version contains supplementary material available at 10.1186/s40035-025-00480-x.

## Background

TAR DNA binding protein 43 (TDP-43) is a highly conserved, ubiquitously expressed nuclear protein involved in various aspects of RNA metabolism, including RNA splicing, transcriptional regulation, and mRNA stabilization [1, 2]. Abnormalities in TDP-43, such as cytoplasmic mislocalization and aggregation, are strongly implicated in the pathogenesis of several neurodegenerative disorders, in particular amyotrophic lateral sclerosis (ALS) and frontotemporal lobar degeneration (FTLD), which are collectively referred to as TDP-43 proteinopathies [3–6]. In these diseases, TDP-43 loses its normal nuclear function and forms pathological aggregates in the neuronal cytoplasm [7], leading to cell dysfunction and death [8]. These common pathological features suggest an important role for TDP-43 in the pathogenesis.

The C-terminus of TDP-43 contains an intrinsically disordered region (IDR) that includes a glycine-rich low complexity domain and a glutamine/asparagine-rich prion-like domain [9]. TDP-43 enhances its function through liquid–liquid phase separation (LLPS) via the IDR [10, 11]. On the other hand, the IDR of TDP-43 has a high condensation tendency due to its simple tertiary structure [12–14]. Since most ALS-related mutations are found in the IDR, it has been hypothesized that the IDR may contribute to the formation of TDP-43 aggregates [15–17]. It has been reported that mutations in the IDR may induce abnormal phase separation or transition and accumulation of TDP-43 aggregates by increasing the binding stability of TDP-43 [18–20].

Despite considerable advances in understanding the role of TDP-43 in neurodegeneration, the exact mechanisms by which TDP-43 aggregates exert their toxic effects remain unclear [21]. Studies have suggested that TDP-43 aggregates interfere with normal cellular processes such as RNA processing and protein translation, disrupt mitochondrial function, and induce oxidative stress [22–28]. However, direct causal links between TDP-43 aggregation and specific neuronal damage have been difficult to establish due to limitations in controlling the temporal and spatial formation of TDP-43 aggregates in living organisms; the TDP-43 aggregates have been often induced by an exposure of extreme stress condition.

**Fig. 1:**
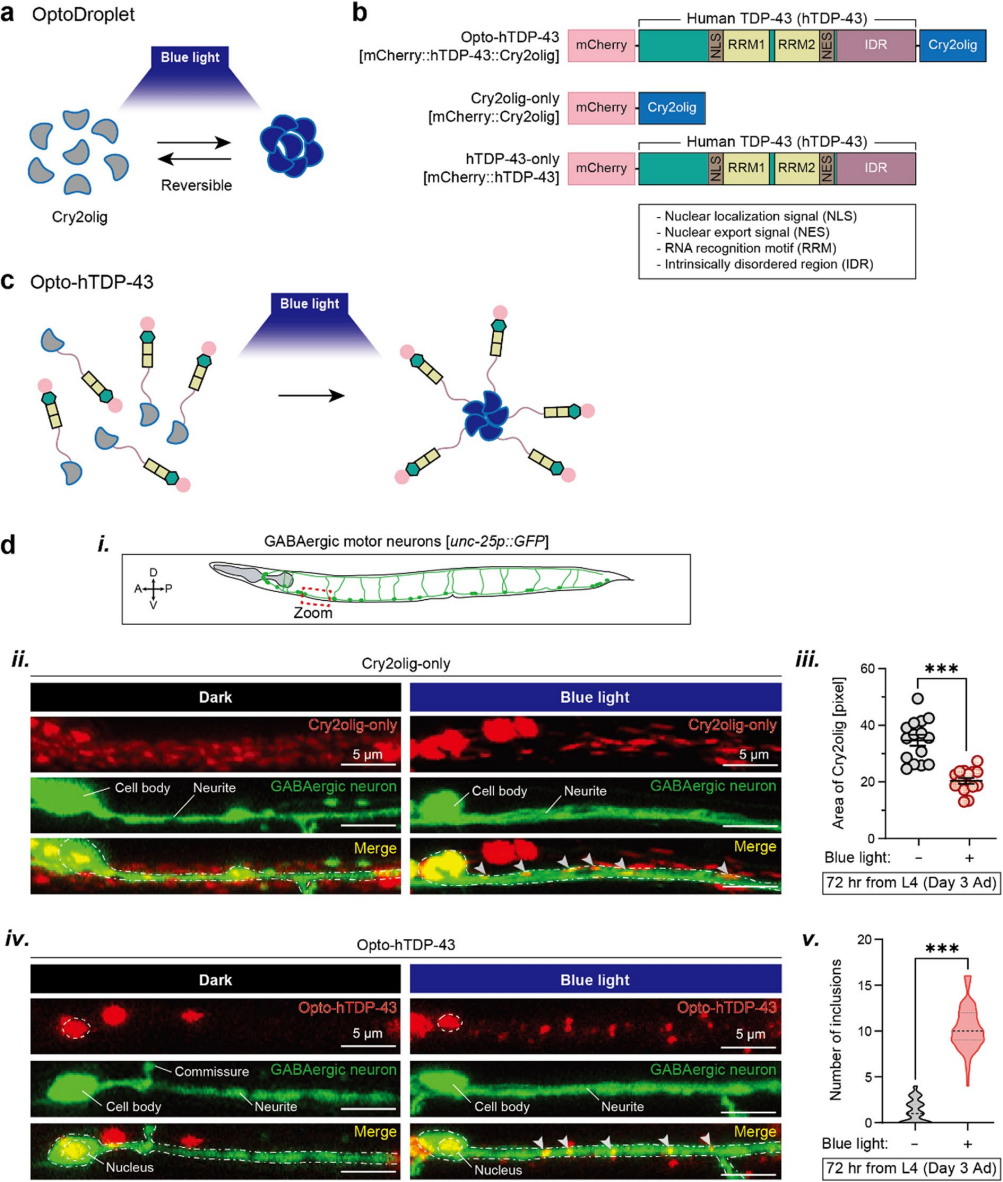
Optogenetic clustering of Cry2olig and opto-hTDP-43 in *C. elegans* neurons. **a** Schematic representation of the optoDroplet system used for inducing clustering of target proteins through blue light exposure. This light-induced clustering is reversible in the dark. **b** Schematic of the transgene constructs used in this study. **c** Schematic of the mCherry::hTDP-43::Cry2olig construct used for optogenetic clustering of hTDP-43. **d** (***i***) GABAergic motor neuron schematic. (***ii***) Confocal images showing the distribution of Cry2olig-only in neurons under dark and blue light conditions. Arrowheads indicate Cry2olig inclusions in the neurite of GABAergic motor neuron. (***iii***) Quantitation of the area of Cry2olig clusters in GABAergic neurons before and after blue light exposure (*n* = 15 worms). Data are presented as mean ± SEM. Unpaired Student’s *t*-test. ***P < 0.001. (***iv***) Confocal images showing the distribution of opto-hTDP-43 in neurons under dark and blue light conditions. Arrowheads indicate opto-hTDP-43 inclusions in the neurite of GABAergic motor neuron. (***v***) Violin graph illustrating the number of opto-hTDP-43 inclusions formed in neurites before and after blue light exposure (*n* = 90). The thick dotted line is the median value. Unpaired Student’s *t*-test. ***P < 0.001. All experiments were independently repeated three times

Optogenetics, which uses light to control cellular processes with high temporal and spatial resolution, provides an innovative approach to study protein aggregation in neurodegenerative diseases [29, 30]. OptoDroplet is a breakthrough technology that induces protein clustering in a light-dependent manner by fusing the target protein to a light-responsive protein domain, such as the Cryptochrome 2 (Cry2) oligomerization domain (Cry2olig) from the plant *Arabidopsis thaliana* [31] (Fig. 1a). The induction of TDP-43 aggregation using this optoDroplet technology has been extended to cell lines and animal models such as fruit flies and zebrafish [32–34]. This method allows precise control over the onset and location of TDP-43 aggregation, providing a unique tool to study the immediate consequences of TDP-43 pathology.

The nematode *C. elegans* provides an excellent model system for these studies due to its transparency, well-characterized nervous system, suitability for genetic manipulation, and real-time imaging [35–39]. In this study, we developed an optogenetic model of TDP-43 proteinopathy in *C. elegans* by generating strains expressing a fusion protein of human TDP-43 and Cry2 (opto-hTDP-43). This approach has allowed us to simulate and observe the effects of TDP-43 aggregation in vivo under controlled experimental conditions. We used this model to elucidate the effects of TDP-43 aggregation on neuronal structure and function, locomotion, and other behavioral phenotypes.

## Materials and methods

### *C. elegans* strains and maintenance

*C. elegans* was handled and cultured using standard method. Worms were maintained at 20 °C on Nematode Growth Medium (NGM) plates seeded with live *Escherichia coli* OP50. The N2 (or Bristol) strain was used as wild-type (WT) *C. elegans*. To synchronize worm cultures, gravid hermaphrodites were bleached using a bleaching solution (1% NaOCl, 0.5 mol/L NaOH). Synchronous first larval (L1) stage groups were obtained by overnight hatching in M9 buffer (42 mmol/L Na_2_HPO_4_, 22 mmol/L KH_2_PO_4_, 8.5 mmol/L NaCl, and 1 mmol/L MgSO_4_) at 20 °C. All strains used in this study are listed in Table S1.

### Plasmids

The PCR-generated fragments of hTDP-43 (NM_007375) cDNA were obtained from the CL6049 strain, and TDP-43 expression vector was constructed using a pCR8/GW/TOPO TA Cloning Kit (Invitrogen, Waltham, MA). In vitro recombination between the hTDP-43 backbone and vector pCZGY1890 (Yishi Jin’s laboratory at the University of California, San Diego, CA) was performed with the Gateway LR Clonase II Enzyme mix (Invitrogen). For pan-neuronal expression of hTDP-43, the *unc-25* promoter sequence in pCZGY1890 was replaced by the *rgef-1* promoter sequence via Gibson assembly (Gibson Assembly Master Mix, New England Biolabs, Ipswich, MA). The reporter mCherry cDNA was obtained from the pCFJ90 plasmid DNA (#19327, Addgene, Watertown, MA). mCherry::hTDP-43 was constructed by adding a PCR-generated mCherry fragment to the N-terminus of hTDP-43 cDNA. An opto-hTDP-43 [mCherry::hTDP-43::Cry2olig] was constructed by fusing Cry2olig (#60032, Addgene) to the C-terminus of mCherry::hTDP-43 (Fig. 1b). Transgenes were generated via Gibson assembly (NEBuilder HiFi DNA Assembly Master Mix, New England Biolabs). The optogenetic control, mCherry::Cry2olig, was generated by deleting hTDP-43 from opto-hTDP-43 using site-specific mutagenesis (Q5 Site-Directed Mutagenesis Mix, New England Biolabs). The sequence of Cry2olig-only [mCherry::Cry2olig] was identical to that of opto-hTDP-43 except for the hTDP-43. All plasmids and primers used in this work are listed in Tables S2 and S3, respectively.

### Transgenic animals

Transgenic animals were generated by microinjecting each DNA plasmid, along with a co-injection marker (*gcy-8p::GFP*, 100 ng/µL) into the N2 germline. All transgenes were microinjected at a concentration of 25 ng/µL. Worms expressing opto-hTDP-43 were exposed to UV gamma radiation to integrate the transgene and these strains were backcrossed five times with N2. The opto-hTDP-43 transgenic animals were then crossed with CZ13799 (*juIs76[unc-25p::GFP]*), CZ631 (*juIs14[acr-2p::GFP]*), or BZ555 (*egIs1[dat-1p::GFP]*), which serve as γ-aminobutyric acid (GABA)-ergic, cholinergic, and dopaminergic neuronal reporter strains, respectively.

### Blue light illumination

For blue light illumination, 100–150 synchronized worms were placed on NGM plates seeded with *E. coli* OP50. Plates were placed 10 cm above a 475-nm LED (W3 Irradiation System, Live Cell Instrument, Namyangju, Republic of Korea), and blue light was continuously illuminated without time intervals. Illumination was performed for 72–120 h, depending on the experiments. Considering the phototoxicity to *C. elegans*, illumination was given at 15% of the total power [40]. Worms were then maintained in the dark on plates wrapped in aluminum foil when necessary.

### Microscopy

Inclusions and neuronal phenotypes in *C. elegans* were observed using a confocal microscope (LSM 710, Zeiss, Oberkochen, Baden-Württemberg, Germany) or an epifluorescence microscope (DM2000, Leica, Wetzlar, Hesse, Germany), with a 40 × or 63 × objective, at wavelengths of 488 nm and 594 nm. All representative images were acquired by confocal microscopy.

### Quantification of inclusions

The blue light condition group was exposed to blue light for 72 h, from the late fourth larval (L4) stage to day 3 of adulthood. To quantify opto-hTDP-43 and Cry2olig inclusions, images were captured using confocal microscopy. Inclusions in neurites extending 20 μm from each cell body were observed in the region encompassing the D-type motor neurons (DD2) and the ventral D-type motor neurons (VD3 and VD4). Inclusion sizes and numbers were analyzed with the Zen Zeiss Lite software. Data were collected from approximately 30 worm samples per group, with results derived from three independent experiments. To analyze Cry2olig dynamics in response to light illumination, fluorescence areas were quantified using the ImageJ software (Version 1.53 k, NIH, Bethesda, MD) based on confocal microscopy images. Data were obtained from at least seven worm samples per group, with results derived from two independent experiments.

### 4’, 6-Diamidino-2-phenylindole (DAPI) staining

For nucleus visualization, *C. elegans* were initially fixed with 4% paraformaldehyde for 15 min followed by three washes with 1 × PBST. To enhance staining efficiency, the fixed worms were incubated in methanol for 30 min. After additional washing steps, the worms were incubated with 500 ng/mL of DAPI for 30 min in dark at room temperature [41].

### Fluorescence recovery after photobleaching (FRAP) analysis

The blue light condition group was exposed to blue light for 72 h, from the L4 stage to day 3 of adulthood. To improve the clarity of light recovery without movement of the worms, we immobilized worms by mounting them in a 40 mmol/L tetramisole drop on a 10% agarose pad. The inclusions of proteins were identified using the 40 × objective of the confocal microscope and image magnified using 4 × digital zoom. Photobleaching was performed by setting a rectangular region of interest (ROI) along either the neurites or the nuclear area, depending on the experimental requirements. We set an ROI in the non-fluorescent area to remove background noise in each control group. Additionally, to control for photobleaching, a reference ROI was set on an adjacent unbleached neurites or cell bodies. Photobleaching was performed using 100% laser (594 nm) power transmission setting for 5 iterations. Fluorescence recovery was monitored at 10-s intervals for 1 min after photobleaching. Fluorescence intensity values after photobleaching were compensated using the reference ROI. Fluorescence values were converted to percentages and the fluorescence intensity value for photobleaching was set to 0%. The data represented were from three independent experiments with at least 4 biological replicates per experiment.

### Neurodegeneration assay

The blue light condition group was exposed to blue light for 96 h, from the late L4 stage to day 4 adulthood. To confirm neurodegeneration, motor neurons labeled with GFP were observed and counted using epifluorescence microscopy or confocal microscopy. GABAergic or cholinergic neurons were considered degenerating when ventral nerve cord gaps were observed in worms. The neuronal defect was classified as follows: ‘Normal’, indicating no neuronal defect; ‘Mild’, one ventral nerve cord breakage; and ‘Severe’, two or more breakages. The data presented were from three independent experiments with at least 15 biological replicates per experiment.

### Motility assay in liquid media (thrashing assay)

The blue light condition group was exposed to blue light for 120 h, from the late L4 stage to day 5 of adulthood. A total of 100–150 worms were harvested from NGM into 1.5 mL tubes and washed twice using M9 buffer. A drop of 40 µL of M9 buffer containing about 20 worms was placed on a 90-mm unseeded NGM plate. After 30 s of adaptation, worms in the M9 buffer drop were randomly selected, and the number of times the head direction changed for 30 s was counted. The thrashing assay was performed in a double-blind manner. The thrashing rate was assessed in ≥ 15 worms per genotype or condition across three independent experiments.

### Motility assay in solid media

The motility assay was adapted from Flavell et al. (2013) [42]. To prepare the plates, *E. coli* OP50 bacteria were cultured overnight in 50 mL of Luria–Bertani (LB) broth at 37 °C. A volume of 500 µL of the culture was evenly spread onto each 35-mm NGM plate, which was left overnight to absorb. The blue light condition group was exposed to blue light for 120 h, starting from the late L4 stage. Immediately following illumination, individual worms were placed on the prepared plates and incubated in the dark for 2 h. A 10 × 10 grid was placed beneath the worm plate. Worm tracks were quantified by counting the number of squares containing tracks, excluding the edges of the grid. A total of 84 squares were analyzed per plate. For each genotype, 5–15 plates were analyzed per experiment, with data derived from three independent experiments.

### Food navigation efficiency assay

Two days before the experiment, *E. coli* OP50 bacteria were cultured in 25 mL of LB broth overnight at 37 °C. The next day, they were concentrated by centrifugation at 6000 rpm and then diluted in 5 mL of M9 buffer. A 50 µL aliquot of concentrate was spotted onto a 60-mm NGM plate. The plate was then incubated overnight at 37 °C to allow liquid to absorb into the agar and the *E. coli* OP50 to grow. The blue light group was exposed for 72 h, from L4 stage to day 3 of adulthood. A total of 100–150 worms were washed twice with the M9 buffer and placed at the farthest bottom of the *E. coli* lawn. The M9 drops were quickly removed with lint-free wipes (Kimwipes). Worms that moved to the lawn were counted every 5 min for a total period of 60 min. The experiment was performed five times independently. To determine the rate of migration to the lawn, the total number of worms on each plate was converted to a percentage.

### Statistical analysis

GraphPad Prism (version 9, Boston, MA) was used for statistical analysis. Statistical significance was defined as *P* < 0.05 for all experiments. Unpaired Student’s *t*-test was performed for comparison of two samples and one-way ANOVA followed by Tukey’s multiple comparison test for multiple comparisons. Fisher’s exact test was used to compare two categorical variables. For comparisons of FRAP and food navigation efficiency assays, two-way ANOVA with Tukey’s multiple comparison test was employed.

## Results

### Optogenetic induction of TDP-43 condensation in *C. elegans* neurons

To study the effects of TDP-43 aggregation in vivo, we used optoDroplet technology to construct optogenetically controllable TDP-43 fused to the Cry2olig domain (Fig. 1b, c). The Cry2olig module, a derivative of Cry2, has been engineered to induce rapid, robust, and reversible protein oligomerization in response to blue light exposure, potentially facilitating LLPS by promoting protein aggregation. In this study, human TDP-43 protein was tagged at its N-terminus with mCherry and at its C-terminus with Cry2olig (Fig. 1b; opto-hTDP-43 [mCherry::hTDP-43::Cry2olig]). Neuronal expression of this construct in *C. elegans* was driven by a pan-neuronal promoter, *rgef-1,* enabling blue light-dependent induction of TDP-43 protein aggregation in neurons. Cry2olig-only [mCherry::Cry2olig] and hTDP-43-only [mCherry::hTDP-43] were also generated to serve as controls (Fig. 1b). We then used qRT-PCR to compare the expression level of opto-hTDP-43 to its *C. elegans* homolog, endogenous TDP-1. The expression level of *TARDBP* (the gene encoding TDP-43) was relatively low, even lower than that of *tdp-1* (Fig. S1a).

We hypothesized that both the opto-hTDP-43 and Cry2olig-only, but not the hTDP-43-only *C. elegans*, would demonstrate condensation in neurons upon blue light exposure due to the light-activated Cry2olig and the transparency of *C. elegans* body (Fig. 1c). We first observed the protein dynamics of each transgenic strain before and after blue light exposure in neurons. To enhance visualization, we labeled the GABAergic neurons with GFP (Fig. 1d*i*). In the Cry2olig-only control, the protein predominantly localized to the cytoplasm of the neuronal cell body (Figs. 1d*ii* and 2a). The protein was initially distributed throughout the neuron in the absence of light (dark conditions), but subsequently formed condensates along the neurite bundles following illumination (Fig. 1d*ii*). As the Cry2olig condensed within the neurite bundles after illumination, the area of fluorescence was significantly reduced (Fig. 1d*iii*).

**Fig. 2:**
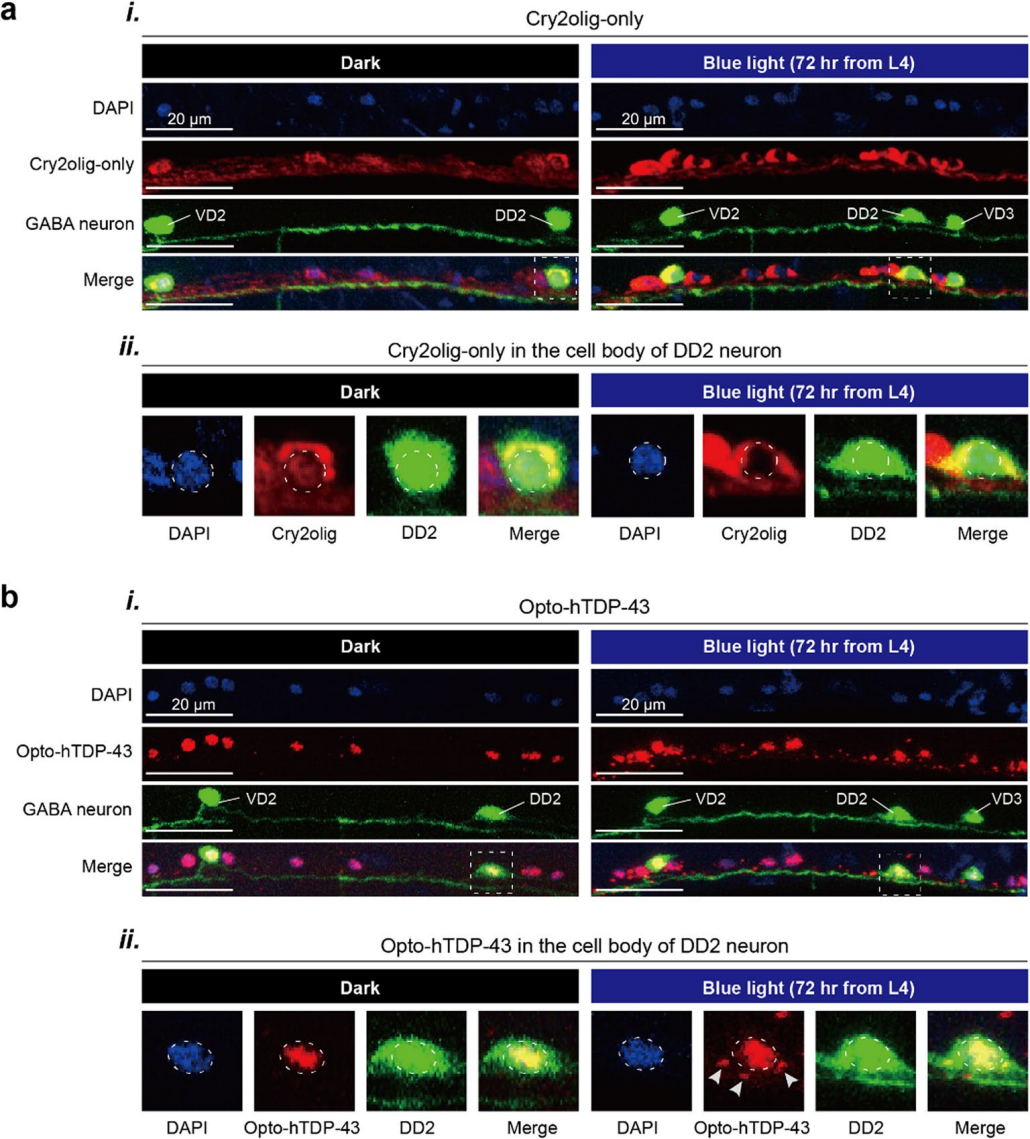
Localization of Cry2olig-only and opto-hTDP-43 proteins in *C. elegans* neurons. **a** Confocal images depicting (***i***) the localization of Cry2olig-only inclusions under dark and blue light conditions and (***ii***) a zoomed-in view of the cell body of the DD2 neuron, with the dotted line outlining the nuclear boundary. **b** Confocal images depicting (***i***) the localization of opto-hTDP-43 inclusions under dark and blue light conditions and (***ii***) a zoomed-in view of the cell body. The arrowheads indicate opto-hTDP-43 inclusions in the cytoplasm

We then examined the localization and dynamics of opto-hTDP-43. In the dark, opto-hTDP-43 was primarily observed in the nucleus, with no condensates observed in the neurite (Figs. 1d*iv* and 2b), showing a localization pattern similar to that of the hTDP-43-only control (Fig. S2a). Upon exposure to blue light, multiple condensates were observed in the nucleus, the cytoplasm of cell bodies, and along neurites (Figs. 1d*iv*, d*v* and 2b). This behavior contrasted with hTDP-43-only control, which did not form condensates in the cytoplasm or neurites, regardless of light exposure (Fig. S2a, b).

Furthermore, we found that the formation of these condensates along neurites depended on the duration of blue light exposure in opto-hTDP-43-expressing worms (Fig. S3). To determine whether the increased number of inclusions resulted from elevated expression of opto-hTDP-43, we performed qRT-PCR to compare the expression levels of opto-hTDP-43 in the presence and absence of inclusions. No significant differences in expression levels were observed (Fig. S1c).

These results demonstrate that the optogenetic modulation induces mislocalization and condensation of TDP-43 in the cell bodies and neurites of *C. elegans* neurons. Collectively, these results suggest that the optogenetic strategy is more effective in clustering TDP-43 than mere overexpression, indicating its potential to model TDP-43 proteinopathy by inducing characteristic mislocalization and inclusions.

### Formation of aggregates by opto-hTDP-43 following blue light exposure

**Fig. 3:**
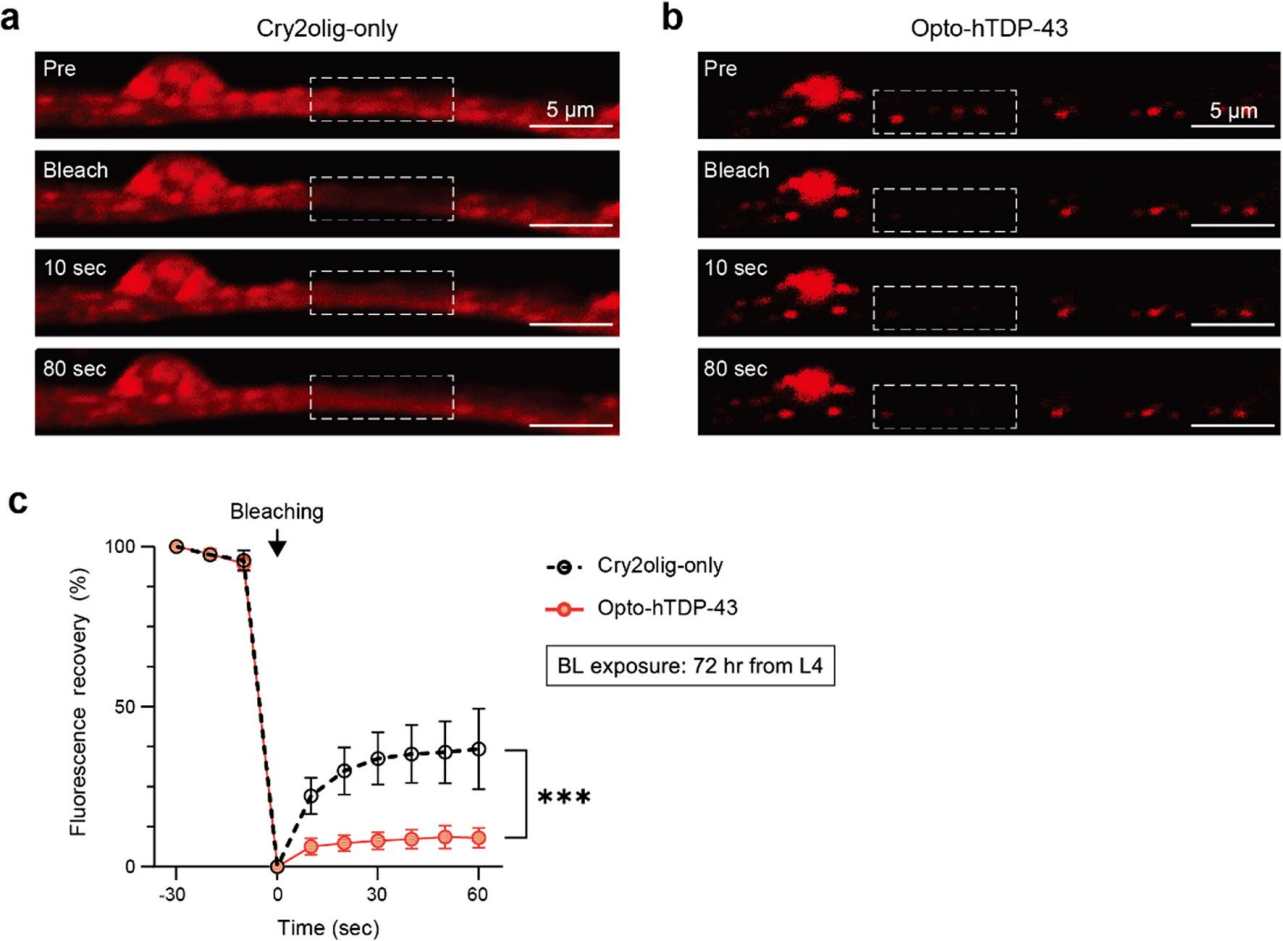
Fluorescence recovery after photobleaching (FRAP) analysis of Cry2olig-only and opto-hTDP-43 inclusions. **a** FRAP analysis of Cry2olig-only in the dark. Confocal images show fluorescence of mCherry::Cry2olig before bleaching (Pre), immediately after bleaching, and at 10 and 80 s post-bleaching in the ventral nerve cord. **b** FRAP analysis of light-induced opto-hTDP-43. Confocal images show fluorescence recovery of mCherry::hTDP-43::Cry2olig. **c** Quantification of fluorescence recovery over time, displayed as percentage recovery (*n* = 15). Quantitative measurements were collected at 10-s intervals, up to 60 s. Data are presented as mean ± SD. Two-way ANOVA. ***P < 0.001. All experiments were independently repeated three times

We next investigated the dynamics of opto-hTDP-43 condensates along neurites induced by blue light exposure with FRAP analysis. The Cry2olig signal exhibited rapid recovery (Fig. 3a); however, the recovery of the opto-hTDP-43 condensate signal was absent after photobleaching (Fig. 3b). The recovery rate of the opto-hTDP-43 condensates was significantly lower than that of Cry2olig (Fig. 3c), indicating stable and possibly irreversible protein aggregates. We also performed FRAP analysis post-illumination on opto-hTDP-43 remaining in the cell body and found a slightly lower recovery rate following blue light exposure (Fig. S4a, b). Together, these results suggest that opto-hTDP-43 condensates transform into stable protein aggregates, likely due to the IDR in TDP-43 after forming condensates through Cry2olig upon exposure to light in *C. elegans* neurons [43].

### Potential insoluble properties of opto-hTDP-43 aggregates

**Fig. 4:**
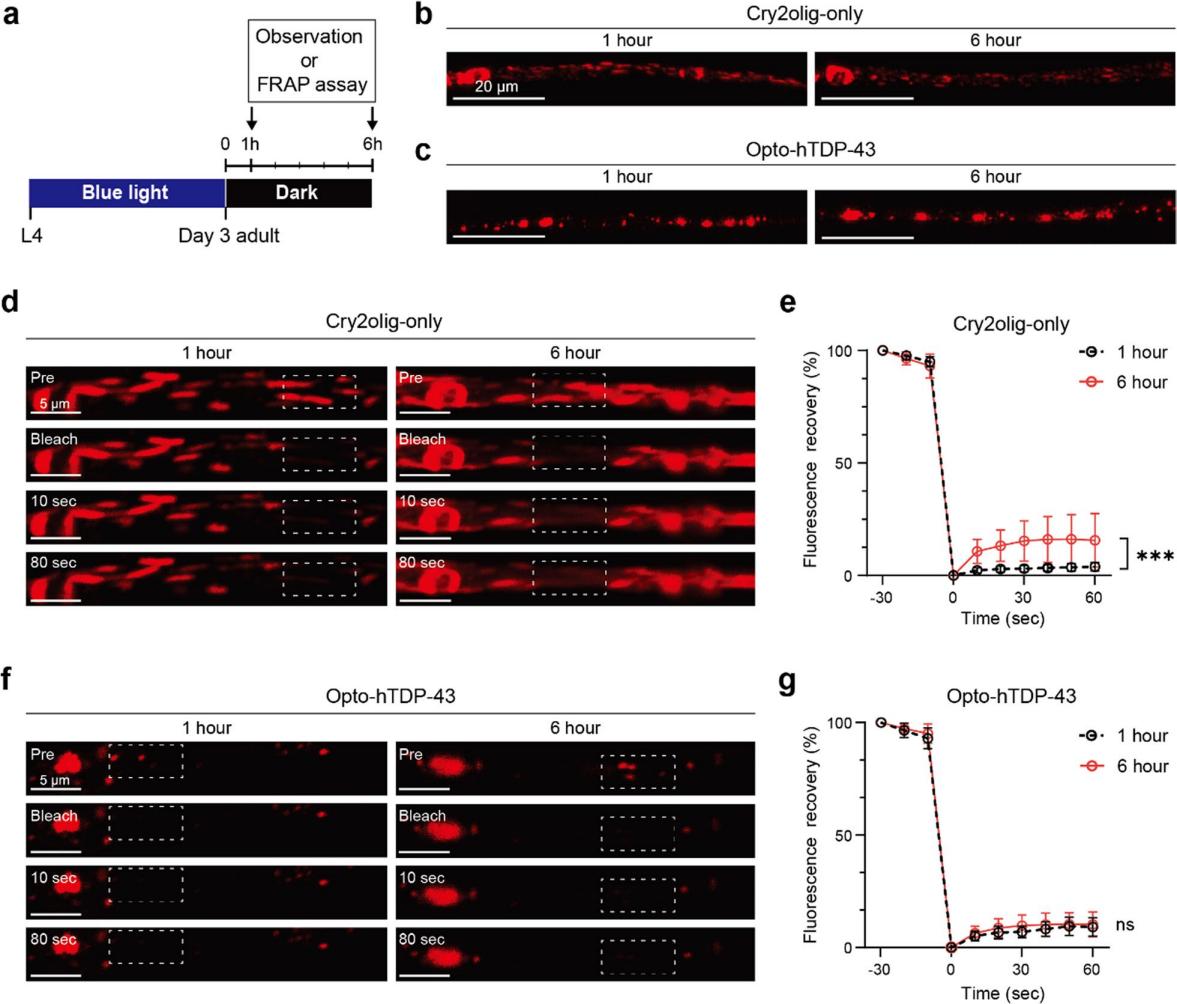
Insolubility analysis of Cry2olig-only and opto-hTDP-43 inclusions. **a** Schematic of timeline depicting the stages of observation and FRAP analysis. **b** Confocal imaging of Cry2olig-only showing the mCherry signal of Cry2olig condensates in dark incubation over the indicated time after illumination. **c** Confocal imaging of opto-hTDP-43 showing the mCherry signal of opto-hTDP-43 aggregates in dark incubation. **d** FRAP analysis of Cry2olig-only at 1 h and 6 h after illumination. Confocal imaging showing the fluorescence recovery of mCherry::Cry2olig before bleaching (Pre), immediately after bleaching, and at 10 and 80 s post-bleaching. Scale bars: 5 μm. **e** Quantification of the fluorescence recovery of the Cry2olig condensates. Quantitative measurements were collected at 10-s intervals, up to 60 s. Graph showing the percentage of fluorescence recovery over time (*n* = 15–20 for each group). Data are presented as mean ± SD. Two-way ANOVA. ***P < 0.001.** f** FRAP analysis of opto-hTDP-43. Confocal imaging showing the fluorescence recovery of mCherry::hTDP-43::Cry2olig. **g** Quantification of the fluorescence recovery of the opto-hTDP-43 aggregates (*n* = 15–20 for each group). Data are presented as mean ± SD. Two-way ANOVA. not significant (ns). All experiments were independently repeated three times for each strain at each time point

In TDP-43 proteinopathies, pathologically aggregated TDP-43 tends to resist degradation after initial aggregation [44, 45]. To determine the persistence of opto-hTDP-43 aggregates, we examined the dynamics of these light-induced structures in comparison to Cry2olig. We exposed late L4 stage worms to blue light for 72 h, then removed the light and observed the proteins 1 h and 6 h later (Fig. 4a). Cry2olig condensates gradually dissipated over time, whereas opto-hTDP-43 condensates remained stable over the same period (Fig. 4b, c). These results demonstrate that opto-hTDP-43 condensates had reduced mobility relative to Cry2olig, suggesting the formation of stable and potentially insoluble aggregates upon exposure to light. However, these opto-hTDP-43 condensates gradually dissolved over an extended period, with dissolution observed after approximately 12 h, eventually returning to a pattern resembling the original pre-light exposure nuclear localization shown in Fig. 1d*iv*.

To further dissect the molecular characteristics of opto-hTDP-43 and Cry2olig condensates, we performed FRAP analysis using the same experimental setup described in Fig. 4a. Cry2olig condensates showed low recovery rate 1 h post-illumination, which increased 6 h post-illumination. This suggests a reversible transition from gel to liquid states in the absence of light (Fig. 4d, e). In contrast, opto-hTDP-43 aggregates showed no recovery of fluorescence after photobleaching, both 1 h and 6 h post-illumination (Fig. 4f, g). These observations suggest that although opto-hTDP-43 aggregates are initially formed by Cry2olig-mediated condensation, they subsequently form stable aggregates primarily due to the TDP-43 protein. Taken together, the properties of opto-hTDP-43 aggregates observed in this model closely resemble those of pathological TDP-43 aggregates found in ALS patients [45].

### GABAergic motor neuron degeneration by opto-hTDP-43 aggregates in *C. elegans*

**Fig. 5:**
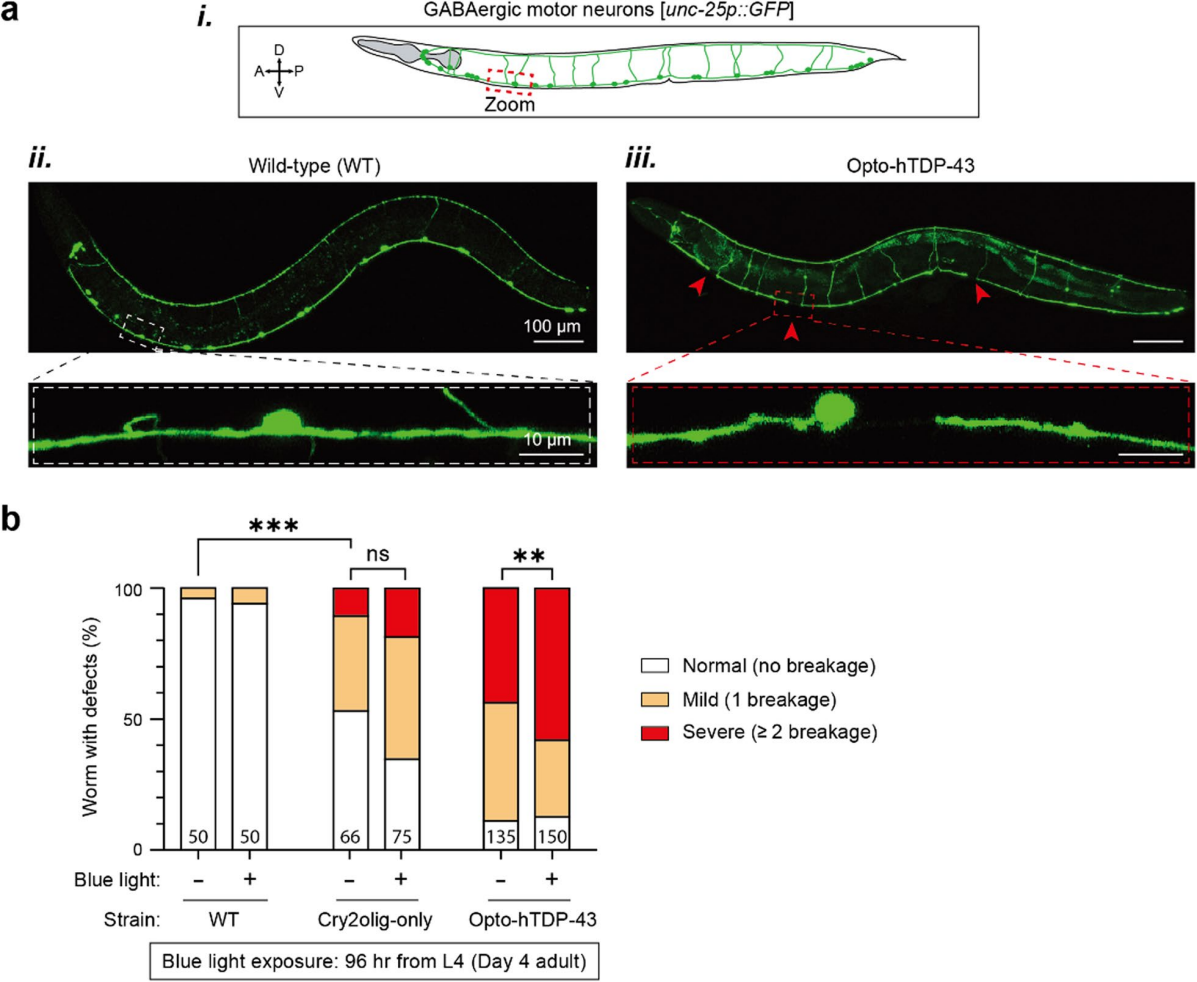
Cellular effects in GABAergic neurons of opto-hTDP-43 expression under blue light stimulation. **a** (***i***) GABAergic motor neuron schematic. Confocal imaging of GABAergic neurons in WT worms (***ii***) and opto-hTDP-43 transgenic worms (***iii***) under blue light stimulation. Red arrowheads indicate gaps in the ventral nerve cord of GABAergic neurons. **b** Quantification of neuronal defects in GABAergic neurons. Bar graph showing the percentage of worms with normal, mild, and severe neuronal defects. The total sample size is shown in the bar. Fisher’s exact test. **P < 0.01, ***P < 0.001, not significant (ns). All experiments were independently repeated three times for each strain under each condition

We demonstrated that opto-hTDP-43 primarily localizes in the nucleus but mislocalizes to the cytoplasm and neurites, forming insoluble aggregates upon light-induced modulation [33, 34]. This mislocalization and aggregation are hallmark features of TDP-43 proteinopathy, which is associated with motor neuron degeneration and dysfunction [8]. To explore whether light-induced opto-hTDP-43 aggregates contribute to neurodegeneration, we analyzed the morphology of GABAergic neurons in *C. elegans* (Fig. 5a*i*).

We hypothesized that the formation of these aggregates would exacerbate neurodegeneration. To test this hypothesis, we quantified neurodegeneration by counting gaps in the ventral nerve cord of GABAergic motor neurons, indicative of neuronal breakage post-illumination. Neurons were categorized as ‘normal’ (no defects), ‘mild’ (one breakage), or ‘severe’ (two or more breakages). Our findings revealed no significant difference in neurodegeneration between Cry2olig-expressing worms with and without light exposure (Fig. 5a*ii*, b). However, significant neurodegeneration was observed in worms expressing opto-hTDP-43 even without light exposure (Fig. 5b), likely due to the exogenous expression of hTDP-43. Remarkably, the severity of neurodegeneration substantially increased with light-induced aggregation of opto-hTDP-43 (Fig. 5a*iii*, b). Thin, branch-like structures appeared to form, potentially following the neuronal break. This pattern suggests that hTDP-43 expression initiates neurodegeneration, while its aggregation accelerates the process. Notably, despite neurite degeneration induced by light-activated opto-hTDP-43 aggregates, the number of GABAergic neuronal cell bodies remained unchanged, indicating that cell death did not occur at the time point of observation.

We further assessed the neurotoxic effects of opto-hTDP-43 on other motor neuron types by examining the morphology of cholinergic motor neurons and dopaminergic neurons. No increased neurodegeneration was observed in these neurons following blue light exposure (Figs. S5 and S6), suggesting that the neurotoxic effects of opto-hTDP-43 are specific to GABAergic neurons [46–48].

### Motility impairment induced by opto-hTDP-43 aggregates in *C. elegans*

**Fig. 6:**
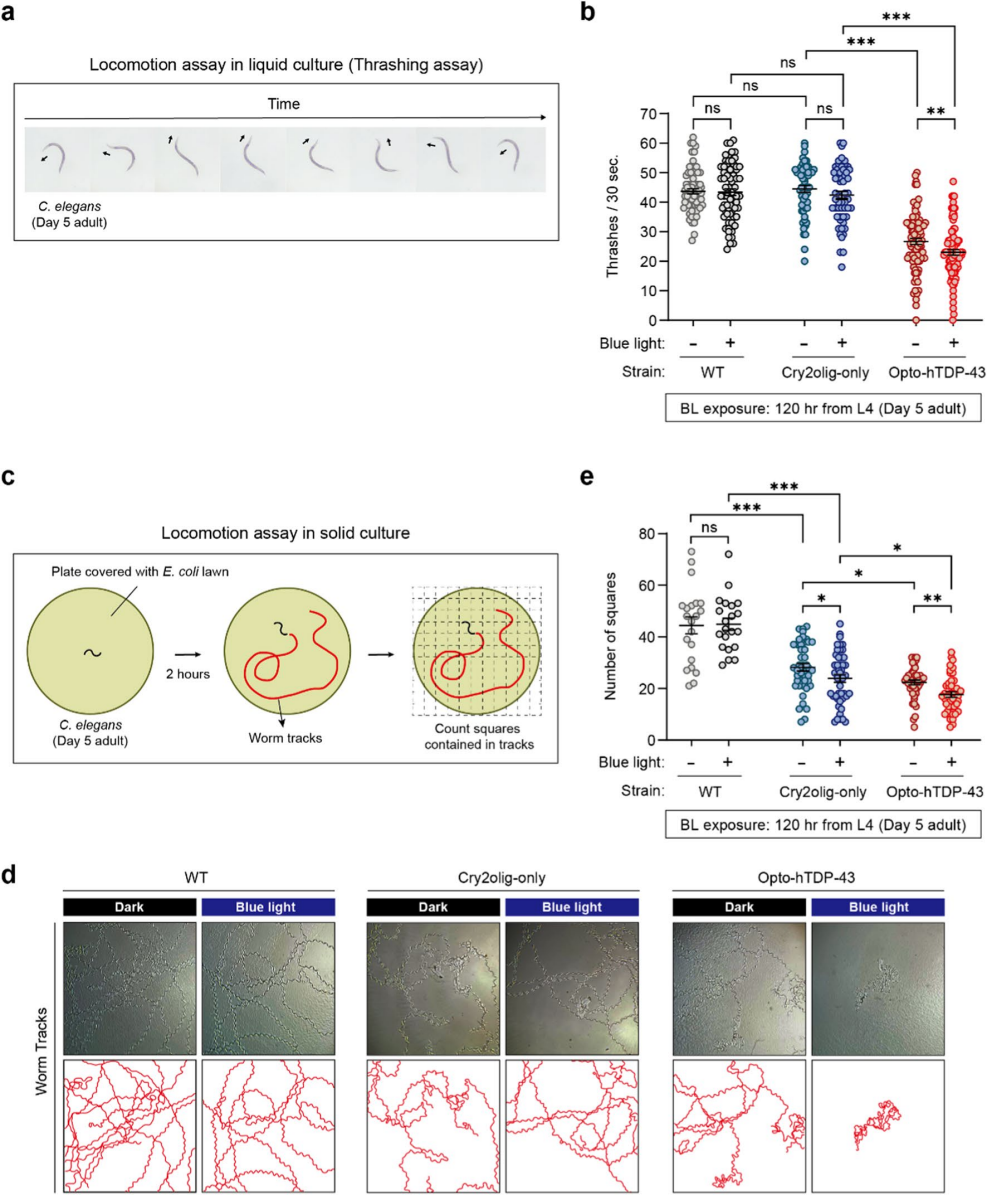
Locomotor effects of opto-hTDP-43 expression under blue light stimulation. **a** Thrashing (lateral swimming) assay. Time-lapse images of worms thrashing in liquid media. The number of thrashes (body bends) per 30 s was recorded to measure the motility of worms in liquid. **b** Quantification of the thrashing behavior. Bar graph showing the average number of thrashes per 30 s for WT, Cry2olig-only and opto-hTDP-43 transgenic worms under dark and blue light conditions. Data are presented as mean ± SEM. One-way ANOVA and unpaired Student’s *t*-test. **P < 0.01, ***P < 0.001, not significant (ns).** c** Worm motility assay in solid media. Schematic representation of the experimental setup for assessing worm motility. Worm tracks were observed on plates coated with *E. coli*, and the number of squares containing tracks was counted to quantify mobility. **d** Images showing the worm tracks under dark and blue light conditions. The images depict the movement patterns of worms on plates containing *E. coli* over 2 h. **e** Quantification of worm tracks with and without blue light. Data are presented as mean ± SEM. One-way ANOVA and unpaired Student’s *t*-test. *P < 0.05, **P < 0.01, ***P < 0.001, not significant (ns)

To investigate the correlation between motor neuron degeneration and motor deficits, we conducted a thrashing assay on *C. elegans* to assess their motility in liquid media. This assay, which involves counting ventral-to-dorsal and dorsal-to-ventral movements, is highly sensitive for detecting motor defects (Fig. 6a) [49]. No phototoxicity was observed in WT (N2) worms exposed to continuous light (Fig. 6b). Similarly, in the transgenic worm strain expressing hTDP-43 (CL6049 strain), no change in mobility was observed following blue light exposure (Fig. S7), indicating that the hTDP-43 protein itself does not exhibit phototoxicity. Additionally, the expression of Cry2olig and the formation of its condensates did not affect mobility (Fig. 6b). However, the expression of opto-hTDP-43 alone induced motor defects, which were further exacerbated by the formation of opto-hTDP-43 aggregates post-illumination (Fig. 6b, Movies S1 and S2).

Further motility analysis on solid media [42, 50] (Fig. 6c) yielded results consistent with those obtained in liquid media, showing no change in mobility in WT and only mild changes in Cry2olig-only worms, indicating no significant phototoxicity (Fig. 6d, e). Consistent with the thrashing assay results, worms expressing opto-hTDP-43 exhibited severe motor defects (Fig. 6d, e). Mild toxicity was also observed in Cry2olig-expressing worms (Fig. 6d, e); however, the absence of specific motor defects from Cry2olig in the thrashing assay suggests that the toxicity may be related to a complex interplay within the *C. elegans* nervous system. Notably, motor defects were significantly more pronounced in worms with opto-hTDP-43 aggregates compared to those with Cry2olig condensates (Fig. 6d, e), indicating a strong association between the formation of opto-hTDP-43 aggregates and motor impairments. Collectively, these findings demonstrate that opto-hTDP-43 aggregates adversely affect motor function and significantly exacerbate motor defects.

### Behavioral impairment induced by opto-hTDP-43 aggregates in *C. elegans*

**Fig. 7:**
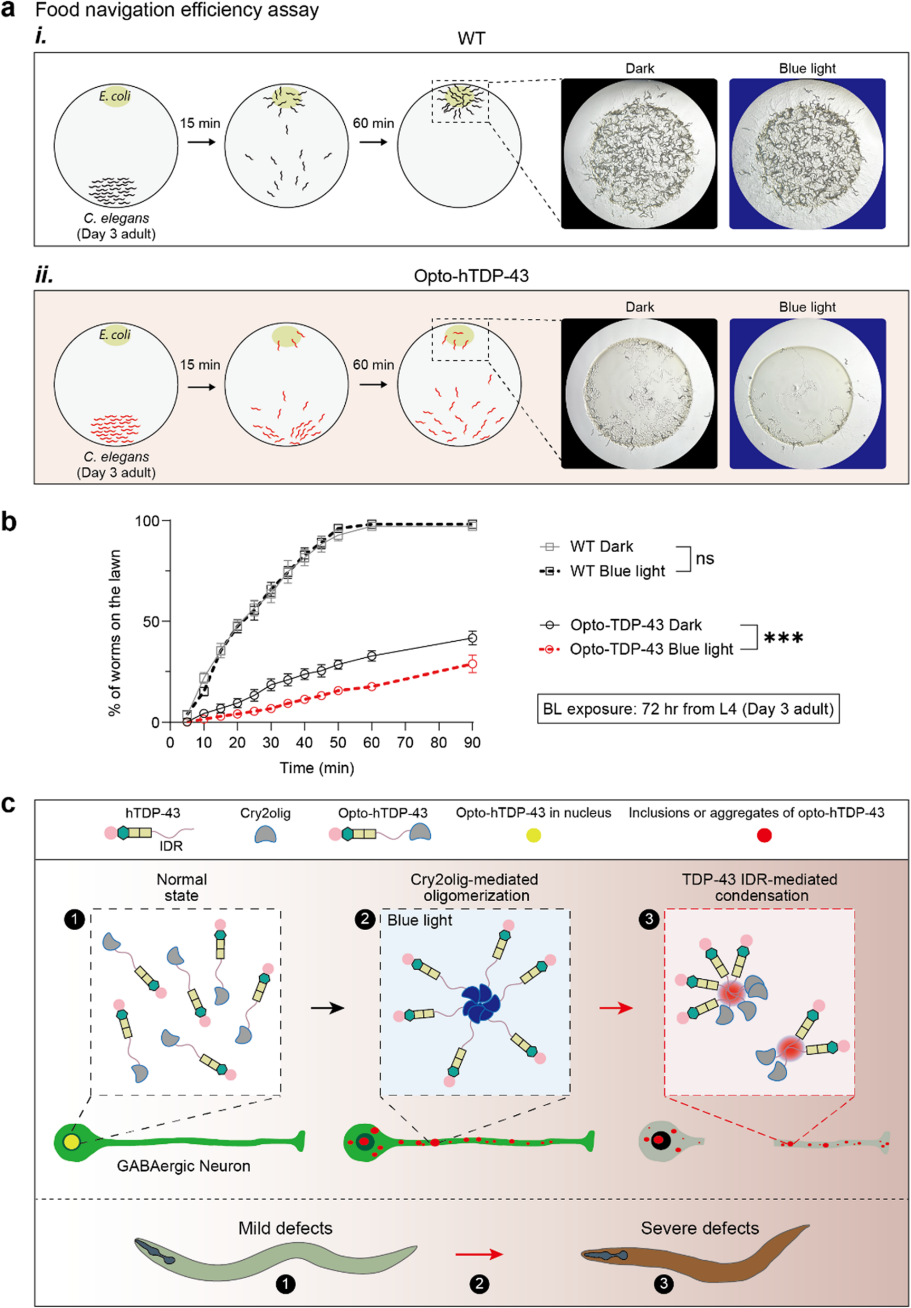
Effects of opto-hTDP-43 expression on food-seeking behavior under blue light stimulation. **a** Schematic representation of the food race assay setup and representative images showing the distribution of WT worms (***i***) and opto-hTDP-43 worms (***ii***) on the bacterial lawn.** b** Quantification of food navigation efficiency assay. Graph showing the percentage of worms on the bacterial lawn over time (*n* > 300 worms). Data are presented as mean ± SEM. Two-way ANOVA. ***P < 0.001, not significant (ns). All experiments were repeated independently five times for each strain.** c** Model of opto-hTDP-43 aggregation and its impact on neuronal defects of *C. elegans.* ① Normal state: in the dark conditions, opto-hTDP-43 proteins are dispersed and predominantly located in the nucleus of neurons. ② Cry2olig-mediated oligomerization: under blue light stimulation, Cry2olig proteins oligomerize and form clusters within the neuron. Opto-hTDP-43 condensates are found in the nucleus, cytoplasm, and neurites of neurons. ③ TDP-43 IDR-mediated condensation: opto-hTDP-43 aggregates persist and exhibit characteristics of insoluble, pathological aggregates. Light-induced opto-hTDP-43 aggregates cause progressive GABAergic neuronal defects, leading to severe defects in the motor and behavioral phenotypes of *C. elegans*

TDP-43 proteinopathy is associated with a range of non-motor symptoms [51]. To investigate whether opto-hTDP-43 expression leads to broader pathological phenotypes beyond motor defects in *C. elegans*, we implemented a food navigation efficiency assay [52]. This assay evaluates the ability of worms to detect and respond to food odors. Adult worms (day 3 after late L4) were placed at a distance from a bacterial lawn, and their movement towards the food source was monitored over time. Fifteen minutes after placement, while WT worms rapidly dispersed toward the odor source, the majority of the opto-hTDP-43-expressing worms remained stationary at the initial spot (Fig. 7a*i*, *ii*). Over an hour, the WT worms efficiently located and migrated onto the bacterial lawn, congregating there almost completely by the 60-min mark (Fig. 7a*i*, b). Conversely, although opto-hTDP-43-expressing worms eventually moved from the starting point, they exhibited difficulty in effectively detecting the bacterial lawn. Furthermore, even when ample time was provided to account for their motility defects, the majority of these worms still failed to reach the food source within 60 min (Fig. 7a*ii*, b). The proportion of opto-hTDP-43-expressing worms that moved to the lawn was significantly lower than that of the WT control, a discrepancy that intensified following light exposure (Fig. 7b). We then tested the mechanosensory function (See Additional file 1 for methods) and found that worms expressing opto-hTDP-43 exhibited more severe mechanosensory defects upon illumination (Fig. S8, Movies S3–S6). Collectively, these results demonstrate that opto-hTDP-43 expression impairs both motor function and sensory processing in *C. elegans*, and these impairments were aggravated when aggregates are formed.

### Shortened lifespan due to opto-hTDP-43 aggregates in *C. elegans*

We examined the impact of opto-hTDP-43 on the lifespan of *C. elegans*. Our results indicate that while WT worms exhibited no significant differences in survival rates under dark versus blue light conditions (Fig. S9a), the survival rate of the opto-hTDP-43 worms under blue light was significantly lower compared to those maintained under dark conditions (Fig. S9b). Specifically, the light-induced opto-hTDP-43 aggregates shortened the average lifespan of *C. elegans* (Fig. S9c). These data underscore the toxic effects of opto-hTDP-43 aggregates induced by light exposure and highlight the potential of light-mediated modulation of protein aggregation as a model to further investigate the mechanisms underlying TDP-43 proteinopathy and its impact on neurodegenerative disease progression.

### TDP-1 does not influence opto-hTDP-43-associated phenotypes

Prior research has suggested that endogenous TDP-1 could influence the behavior or stability of TDP-43 aggregates [53, 54]. To investigate this, we first examined whether the expression of opto-hTDP-43 affects endogenous *tdp-1*. Using qRT-PCR, we compared the expression level of *tdp-1* in WT and opto-hTDP-43 worms. The results showed that opto-hTDP-43 expression did not alter *tdp-1* expression levels (Fig. S1b). Next, we explored if endogenous TDP-1 function influences the neurotoxicity observed in the opto-hTDP-43 model. To this end, we generated an opto-hTDP-43 strain in a *tdp-1* loss-of-function (lf) mutant background to assess whether the absence of TDP-1 impacts opto-hTDP-43 aggregation dynamics or associated pathology. In the *tdp-1(lf)* background, there was no difference in inclusion formation compared to opto-hTDP-43 worms after blue light exposure (Fig. S10a). Additionally, the thrashing assay revealed no significant changes in motility in the absence of TDP-1 (Fig. S10b). These findings indicate that the presence of TDP-1 does not significantly influence inclusion formation or the neurotoxic phenotypes associated with opto-hTDP-43 aggregation.

## Discussions

In this study, we employed an innovative optogenetic approach to induce and examine TDP-43 aggregation in *C. elegans*, providing valuable insights into the neurodegenerative consequences associated with TDP-43 proteinopathy. By developing and utilizing the opto-hTDP-43 model of *C. elegans*, we were able to control the temporal and spatial dynamics of TDP-43 aggregation and directly observe its effects on neuronal health and organismal behavior (Fig. 7c). Our findings have significant implications for understanding the mechanisms of TDP-43-related neurodegeneration and for developing potential therapeutic strategies.

### Direct link between TDP-43 aggregation and neurodegeneration

The results of this study demonstrate a clear correlation between optogenetically induced TDP-43 aggregation in neuronal cells and distinct neurodegenerative changes, particularly in GABAergic motor neurons. The increased severity of neurodegeneration observed with light-induced aggregation, compared to non-aggregated controls, confirms the toxic role of TDP-43 aggregates. Notably, we were able to induce TDP-43 aggregate formation without application of extreme stresses (Figs. 1 and 2), thereby demonstrating that TDP-43 aggregates accelerate the degeneration of *C. elegans* motor neurons and cause motor and behavioral defects similar to those observed in ALS patients.

### Neurotoxicity of TDP-43 aggregates

This study demonstrates that the molecular dynamics of opto-hTDP-43 condensates formed after illumination are reduced (Fig. 3), indicating that the TDP-43 condensates in *C. elegans* neurons convert into irreversible aggregates. These TDP-43 aggregates persist in the cell body and neurites compared to Cry2olig condensates (Fig. 4). These data suggest that the opto-hTDP-43 initially forms condensates through the Cry2olig domain and subsequently aggregates through the domains within TDP-43, likely IDRs. It is hypothesized that when TDP-43 aggregates have formed, their irreversible properties are maintained due to interactions among the IDRs of TDP-43 triggered by the Cry2olig recruitment [9, 18, 19]. Consequently, the opto-hTDP-43 aggregates exhibit molecular characteristics similar to those observed in pathological TDP-43 in ALS patients [43].

Notably, our findings indicate that the formation of opto-hTDP-43 aggregates results in more severe neurodegeneration (Fig. 5). These results are consistent with findings in zebrafish employing light-inducible Cry2 fused with zebrafish TDP-43 in spinal or motor neurons and in *Drosophila* utilizing a comparable optoTDP-43 fusion protein in motor neurons [33, 34]. This indicates that the neurotoxicity of TDP-43 aggregation is evolutionarily conserved. Notably, the neurodegenerative effects of TDP-43 aggregates were more pronounced in GABAergic neurons, whereas cholinergic or dopaminergic neurons exhibited resilience under similar conditions [48] (Figs. 5, S5 and S6). This differential vulnerability highlights the possibility of cell-type-specific factors influencing susceptibility to TDP-43 toxicity [46, 47]. Understanding these factors could provide key insights into the varied clinical manifestations of TDP-43 proteinopathies in humans, such as ALS and FTLD, and might help identify vulnerable neuronal populations in these diseases.

### Behavioral impacts of TDP-43 aggregation

The behavioral assays conducted in this study revealed significant impairments in both motility and food race behavior in worms expressing opto-hTDP-43 (Figs. 6 and 7). These behavioral changes are particularly notable as they offer a functional readout of the neurological impact of TDP-43 aggregates. The correlation between aggregate formation and behavioral deficits underscores the potential for TDP-43 aggregates to disrupt neural circuits and motor functions, reflecting the clinical symptoms observed in TDP-43 proteinopathy patients [6, 55].

### Optogenetics as a tool for modeling neurodegenerative diseases

The use of optogenetics in this study highlights its potential as a powerful tool for modeling neurodegenerative diseases and investigating the dynamics of disease-associated proteins [30, 31]. Neurodegenerative diseases such as Alzheimer’s disease, Parkinson’s disease, Huntington’s disease, and ALS have been reported to have similar pathological mechanisms, including protein aggregation [56, 57]. However, the precise pathogenesis of protein aggregation remains unidentified. The optogenetic technique can serve as a valuable system to modulate protein aggregation in neurodegenerative diseases [31]. This approach allows for controlled induction of protein aggregates, facilitating studies of the immediate effects of such aggregates on cellular functions and rapid screening of potential therapeutic interventions.

### Therapeutic implications of TDP-43 modulation

The ability to induce and reverse TDP-43 aggregation in a controlled manner represents a promising avenue for therapeutic intervention. By elucidating the pathways disrupted by TDP-43 aggregation, targeted therapies can be developed to either prevent initial aggregation or promote aggregate clearance. Moreover, the optogenetic model employed in this study could serve as a robust platform for screening potential drugs capable of modulating TDP-43 pathology [58, 59]. Our model, which combines the biological advantages of *C. elegans* with the benefits of optogenetic modulation, enables effective screening of therapeutic agents that suppress TDP-43 aggregation and neuronal damage, as evidenced by behavioral readouts [60, 61]. The high-throughput capabilities of *C. elegans* facilitate rapid screening of compounds inhibiting aggregation or neurodegeneration, providing insights into therapeutic effects before translation to higher organisms.

### Limitations and future directions

While this study provides crucial insights, it also underscores several areas for further investigation. The mechanisms through which TDP-43 aggregates exert their toxic effects require further elucidation. Future studies should explore the molecular pathways affected by TDP-43 aggregation, potentially utilizing transcriptomic and proteomic approaches. Furthermore, extending these investigations to mammalian models could validate the findings and evaluate their relevance to human disease.

## Conclusions

The optogenetic *C. elegans* model of TDP-43 proteinopathy harnesses optogenetics to precisely replicate TDP-43 aggregation, yielding insights into its pathogenic involvement in neurodegenerative diseases like ALS and FTLD. These findings not only deepen our understanding of TDP-43 proteinopathy but also offer promising avenues for novel therapeutic interventions to mitigate or halt the progression of associated disorders.

## Supplementary Information

**Supplementary file 1**. **Supplementary Materials and Methods**. **Figure S1**. mRNA expression levels of *C. elegans* *tdp-1* and human *TARDBP*. **Figure S2**. Localization of hTDP-43 protein in *C. elegans *neurons. **Figure S3**. Time-dependent formation of Cry2olig-only and opto-hTDP-43 inclusions in *C. elegans* neurons under blue light exposure. **Figure S4**. Fluorescence recovery after photobleaching (FRAP) analysis of nuclear opto hTDP-43 inclusions. **Figure S5**. Cellular effects in cholinergic neurons of opto-hTDP-43 expression under blue light stimulation. **Figure S6**. Cellular effects in dopaminergic neurons of opto-hTDP-43 expression under blue light stimulation. **Figure S7**. Assessment of phototoxicity on hTDP-43 protein. **Figure S8**. Mechanosensory defects in opto-hTDP-43-expressing worms under blue light stimulation. **Figure S9**. Lifespan-reducing effects of opto-hTDP-43 expression under blue light stimulation. **Figure S10**. Lack of TDP-1 shows limited effect on motor function in opto-hTDP-43 worms. **Table S1**. *C. elegans *strains used in this study. **Table S2**. Plasmids used in this study. **Table S3**. Primers used in this study. 

**Supplementary file 2**. **Movie S1**, associated with Figure 5b, showing the thrashing behavior of opto-hTDP-43 worms. Movie S1 depicts worms cultured in dark conditions.

**Supplementary file 3**. **Movie S2**, associated with Figure 5b, showing the thrashing behavior of opto-hTDP-43 worms. Movie S2 shows worms exposed to 120 h of blue light starting from the L4 stage.

**Supplementary file 4**. **Movie S3**, associated with Figure S8, showing the response behavior of wild-type worms to gentle touch. Movie S3 depicts worms cultured in dark conditions.

**Supplementary file 5**. **Movie S4**, associated with Figure S8, showing the response behavior of wild-type worms to gentle touch. Movie S4 shows worms exposed to 72 h of blue light starting from the L4 stage.

**Supplementary file 6**. **Movie S5**, associated with Figure S8, showing the response behavior of opto-hTDP-43 worms to gentle touch. Movie S5 depicts worms cultured in dark conditions.

**Supplementary file 7**. **Movie S6**, associated with Figure S8, showing the response behavior of opto-hTDP-43 worms to gentle touch. Movie S6 shows worms exposed to 72 h of blue light starting from the L4 stage.

## Data Availability

The data generated and analyzed as a part of this study are included within this article (as well as supplementary additional files). Additional datasets supporting the findings of this study are available from the corresponding author upon reasonable request.

## Abbreviations

ALS: Amyotrophic lateral sclerosis
Cry2: Cryptochrome 2
Cry2olig: Cryptochrome 2 oligomerization domain
FRAP: Fluorescence recovery after photobleaching
FTLD: Frontotemporal lobar degeneration
GABA: γ-Aminobutyric acid
IDR: Intrinsically disordered region
LLPS: Liquid-liquid phase separation
Opto-hTDP-43: Optogenetic human TDP-43
TDP-43: Transactive response DNA-binding protein 43 kDa
