# Supplementary material for: Optogenetic induction of TDP-43 aggregation impairs neuronal integrity and behavior in Caenorhabditis elegans
Source: Transl Neurodegener. 2025 Apr 16;14:20. doi: 10.1186/s40035-025-00480-x (PMC12001655; doi:10.1186/s40035-025-00480-x)
Supplement: Supplementary file 1 — Supplementary Materials and Methods. Figure S1. mRNA expression levels of C. elegans tdp-1 and human TARDBP. Figure S2. Localization of hTDP-43 protein in C. elegans neurons. Figure S3. Time-dependent formation of Cry2olig-only and opto-hTDP-43 inclusions in C. elegans neurons under blue light exposure. Figure S4. Fluorescence recovery after photobleaching (FRAP) analysis of nuclear opto hTDP-43 inclusions. Figure S5. Cellular effects in cholinergic neurons of opto-hTDP-43 expression under blue light stimulation. Figure S6. Cellular effects in dopaminergic neurons of opto-hTDP-43 expression under blue light stimulation. Figure S7. Assessment of phototoxicity on hTDP-43 protein. Figure S8. Mechanosensory defects in opto-hTDP-43-expressing worms under blue light stimulation. Figure S9. Lifespan-reducing effects of opto-hTDP-43 expression under blue light stimulation. Figure S10. Lack of TDP-1 shows limited effect on motor function in opto-hTDP-43 worms. Table S1. C. elegans strains used in this study. Table S2. Plasmids used in this study. Table S3. Primers used in this study. [file 40035_2025_480_MOESM1_ESM.pdf]

Translational Neurodegeneration

Supplementary Information

Optogenetic induction of TDP-43 aggregation impairs neuronal integrity and behavior in *Caenorhabditis elegans*

Kyung Hwan Park[1], Euihyeon Yu[1], Sooji Choi[1], Sangyeong Kim[1], Chanbin Park[2,3], J. Eugene Lee[2,3] and Kyung Won Kim[1, #]

[#] Corresponding author: *Kyung Won Kim* kwkim@hallym.ac.kr (K. Kim).

Supplementary Materials and Methods

Quantitative RT-PCR (qRT-PCR)

The blue light condition group was exposed to blue light for 24 hours, from the late fourth larval (L4) stage until day 1 of adulthood. Total RNA was extracted using Trizol reagent (Ambion). Subsequently, 1 μg of RNA was reverse transcribed into cDNA using the ReverTra Ace qPCR RT Master Mix with gDNA Remover kit (Toyobo). Quantitative PCR was conducted using KOD SYBR qPCR Mix (Toyobo) on a Bioneer Exicycler 96 system. Relative RNA expression levels of *tdp-1* and human *TARDBP* were quantified using RQ ($2^{-\Delta\Delta Ct}$) method, with normalization to the housekeeping gene glyceraldehyde 3-phosphate dehydrogenase (*gpd-1/4*). The primer sequences used for qRT-PCR analysis are listed in Table S3. All experiments were conducted with three biological replicates, each including two technical replicates.

Quantification of inclusions

Three independent blue light condition groups were established, with exposure durations of 24 hours, 96 hours, and 120 hours, starting from the L4 stage. Opto-hTDP-43 inclusions were analyzed in neurites extending bilaterally from the D-type motor neuron (DD2) to the ventral D-type motor neurons (VD3 or VD4). Inclusion sizes were compared using Zen Zeiss Lite software, which was used to define a region of interest (ROI) and quantify the inclusion area. Data were obtained from 15 worm samples per group, with results derived from two independent experiments.

Dopaminergic neurodegeneration assay

The blue light condition group was exposed to blue light for 96 hours, from the late L4 stage to day 4 of adulthood. Dopaminergic neurons were classified as degenerative based on the presence of blebbing in the dendrite of cephalic sensilla (CEP) neurons[1]. Dopaminergic neurodegeneration was analyzed using two criteria: normal and mild (indicated by the presence of dendritic blebbing). The data were obtained from 60 worms per condition across two independent experiments. Representative images were acquired using confocal microscopy.

Mechanosensory assay

The touch sensitivity of *C. elegans* was assessed using the gentle touch assay[2]. The blue light condition group was exposed to blue light for 72 hours, from the L4 stage to day 3 of adulthood.

For each assay, 10–20 worms were transferred to fresh, non-seeded NGM plates and acclimated for 20 minutes. Touch stimuli were applied using a sterilized eyebrow hair that was cleaned with 70% ethanol and air-dried completely between assays to prevent ethanol-induced effects. The eyebrow tip was gently stroked across the posterior pharyngeal region of each animal, with 10 consecutive touches applied at 10-second intervals. Behavioral responses to each touch were recorded and classified into three categories: backward movement, head-only movement, or no response. Touch sensitivity was evaluated based on the following criteria. 'full response' was defined as backward movement observed in all trials, 'partial response' indicated at least one trial with head-only movement or no response, and 'no response' was assigned when no movement was observed in any trial.

Lifespan analysis

For each experiment, 50–60 synchronized embryos were placed on NGM plates seeded with *E. coli* OP50. All lifespan analyses were performed at 20 °C. In the blue light condition group, exposure was continued until all worms had died. Worms were transferred to fresh NGM plates seeded with OP50 every 2–3 days using a sterilized platinum wire. Viability was also scored every 2–3 days. A worm was considered dead if it did not respond to gentle touching with the platinum wire. Worms that were missing, had crawled off the plate, or were buried in the medium were censored from the analysis. The log-rank test in OASIS2 software was used for statistical comparison of lifespans[3].

References

1. Lee, Y., S. Choi, and K.W. Kim, *Dithianon exposure induces dopaminergic neurotoxicity in Caenorhabditis elegans.* Ecotoxicol Environ Saf, 2023. **255**: p. 114752.

2. Chalfie, M., et al., *Assaying mechanosensation.* WormBook, 2014.

3. Han, S.K., et al., *OASIS 2: online application for survival analysis 2 with features for the analysis of maximal lifespan and healthspan in aging research.* Oncotarget, 2016. **7**(35): p. 56147-56152.

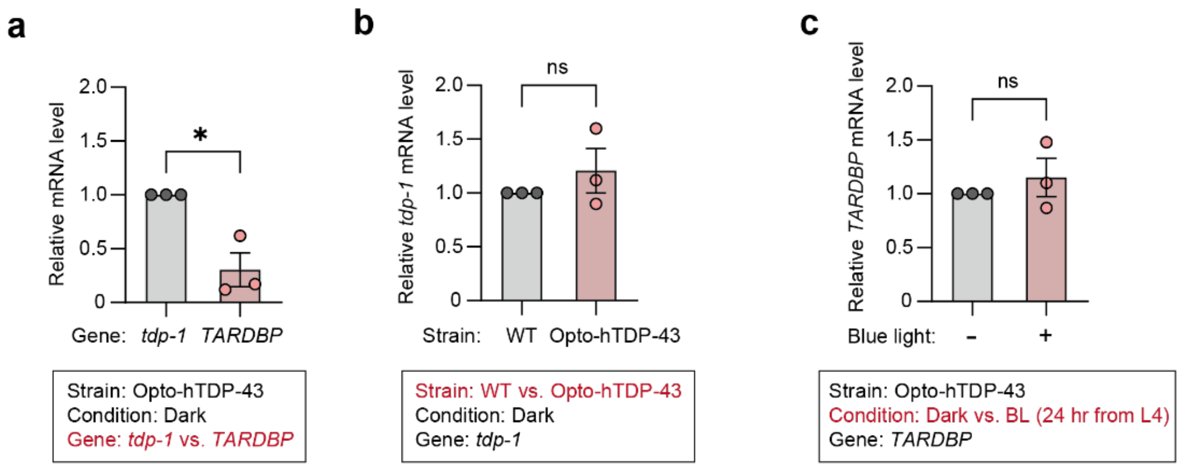

Figure S1. mRNA expression levels of *C. elegans tdp-1* and human *TARDBP*

(**a**) Relative quantification of qRT-PCR showing the mRNA abundance of *tdp-1* and human *TARDBP* (gene encoding TDP-43) in opto-hTDP-43-expressing worm strain. (**b**) Relative quantification of qRT-PCR showing mRNA abundance of *tdp-1* in WT (N2) and opto-hTDP-43 strain. (**c**) Relative quantification of qRT-PCR showing mRNA abundance of *TARDBP* in opto-hTDP-43 strain under dark and blue light illumination conditions. All bar graphs are normalized to *gpd-1/4* (housekeeping genes). Data are represented as mean ± SEM of three biological replications. Unpaired Student's t-test; **P* <0.05, not significant (ns).

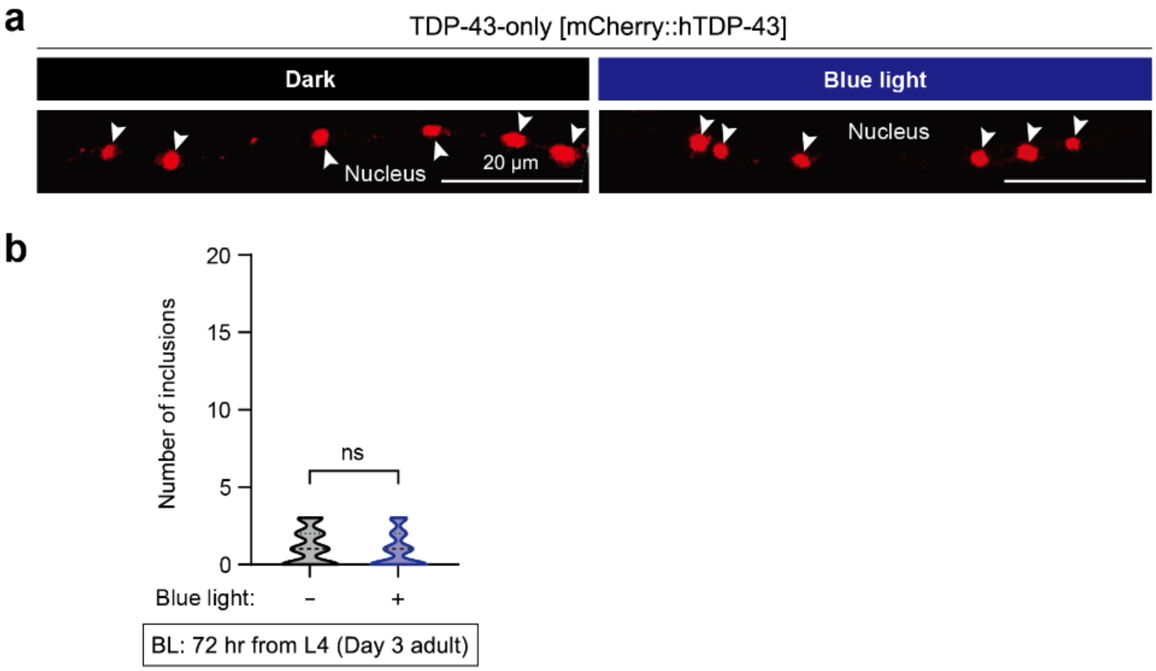

Figure S2. Localization of hTDP-43 protein in *C. elegans* neurons
(**a**) Confocal imaging showing the localization and distribution of hTDP-43-only [mCherry::hTDP-43] inclusions under dark and blue light conditions. (**b**) Quantification of hTDP-43 inclusions. Violin graph showing the number of hTDP-43 inclusions under dark and blue light conditions (n = 90). The thick dotted line is the median value. Unpaired Student's t-test. not significant (ns). All experiments were repeated independently three times.

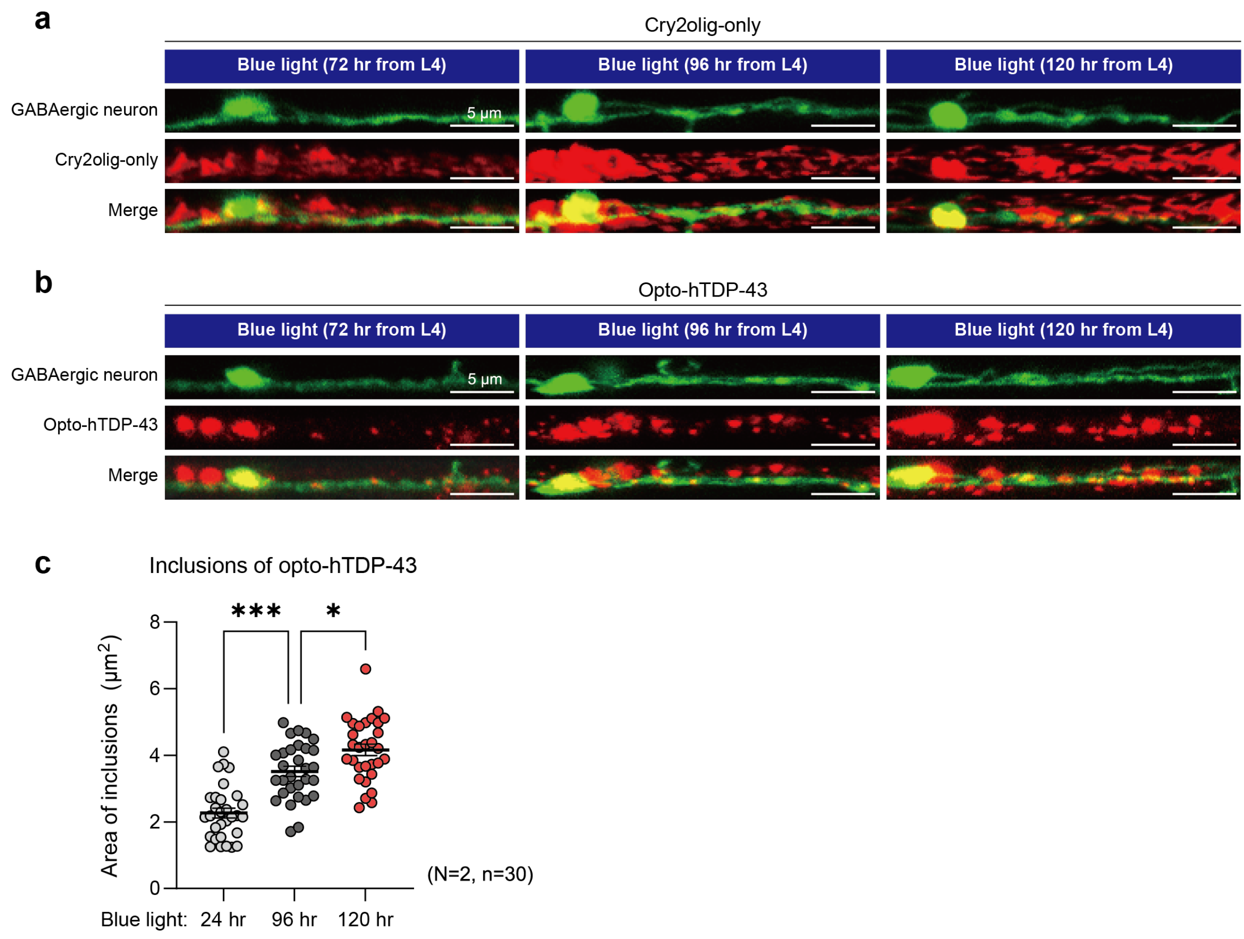

Figure S3. Time-dependent formation of Cry2olig-only and opto-hTDP-43 inclusions in *C. elegans* neurons under blue light exposure

(**a**) Confocal images showing Cry2olig-only [mCherry::Cry2olig]. (**b**) Confocal images showing opto-hTDP-43 [mCherry::hTDP-43::Cry2olig]. (**c**) Quantitation of opto-hTDP-43 inclusions over blue light exposure time (n = 30). Experiments were independently repeated twice. Data are presented as mean ± SEM. One-way ANOVA. *$P < 0.05$; ***$P < 0.001$.

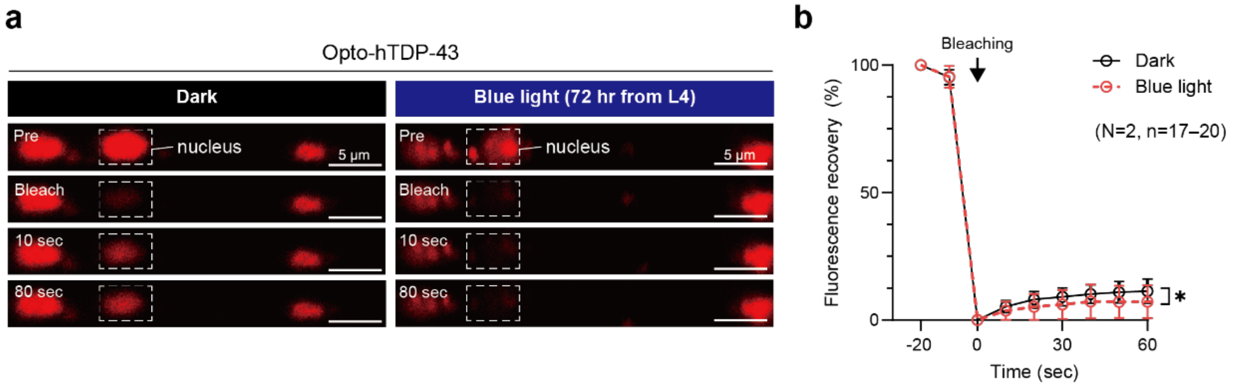

Figure S4. Fluorescence recovery after photobleaching (FRAP) analysis of nuclear opto-hTDP-43 inclusions.

(**a**) FRAP analysis of opto-hTDP-43 in GABAergic neurons. Confocal images show fluorescence recovery of mCherry::hTDP-43::Cry2olig before bleaching (Pre), immediately after bleaching, and at 10 and 80 seconds post-bleaching in the nucleus. Scale bar: 5 µm. (**b**) Quantitation of fluorescence recovery over time, displayed as percentage recovery (n = 17 and 20). Quantitative measurements were collected at 10-second intervals, up to 60 seconds. Data are presented as mean ± SD. Two-way ANOVA. *$P < 0.05$. All experiments were independently repeated twice.

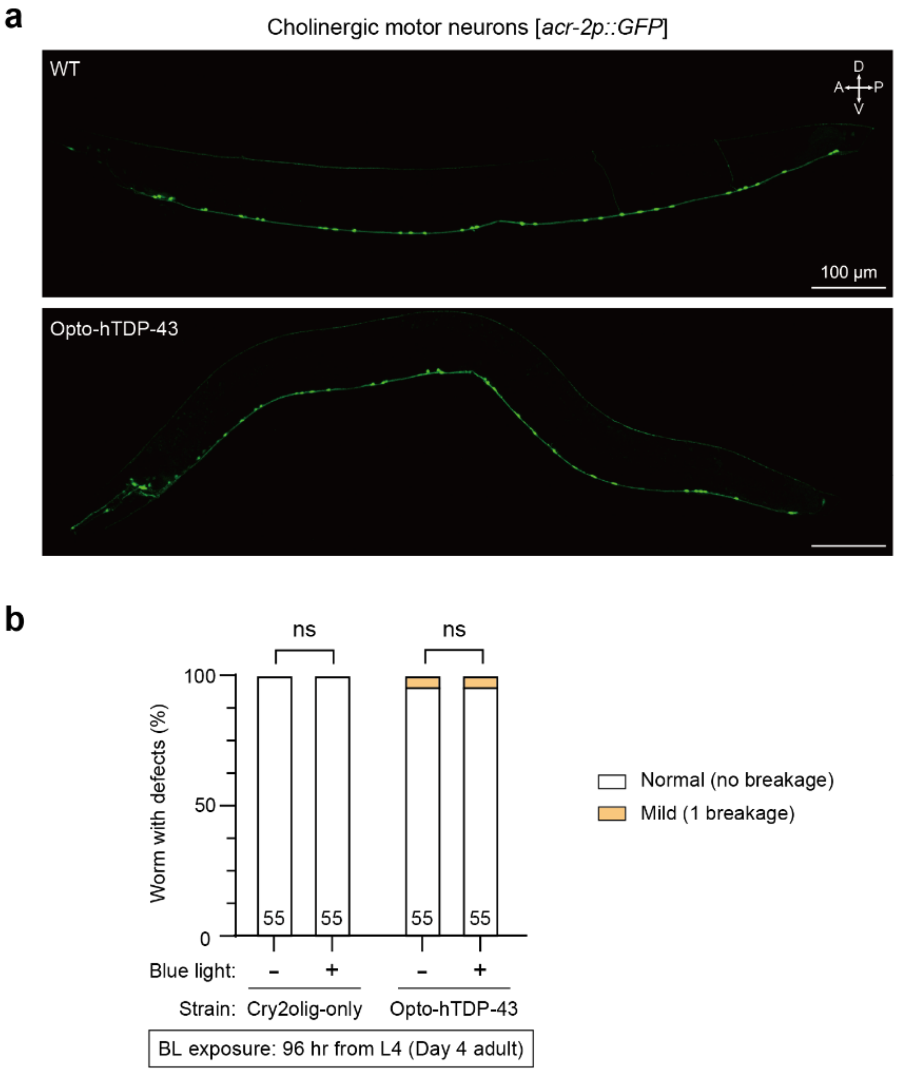

Figure S5. Cellular effects in cholinergic neurons of opto-hTDP-43 expression under blue light stimulation.

(**a**) Confocal images of cholinergic neurons in WT and opto-hTDP-43 transgenic worms under blue light stimulation. (**b**) Quantification of neuronal defects in cholinergic neurons. Bar graph showing the percentage of worms with normal and mild neuronal defects in cholinergic neurons under dark and blue light conditions. The total sample size is shown in the bar. Fisher's exact test; not significant (ns). All experiments were repeated independently three times.

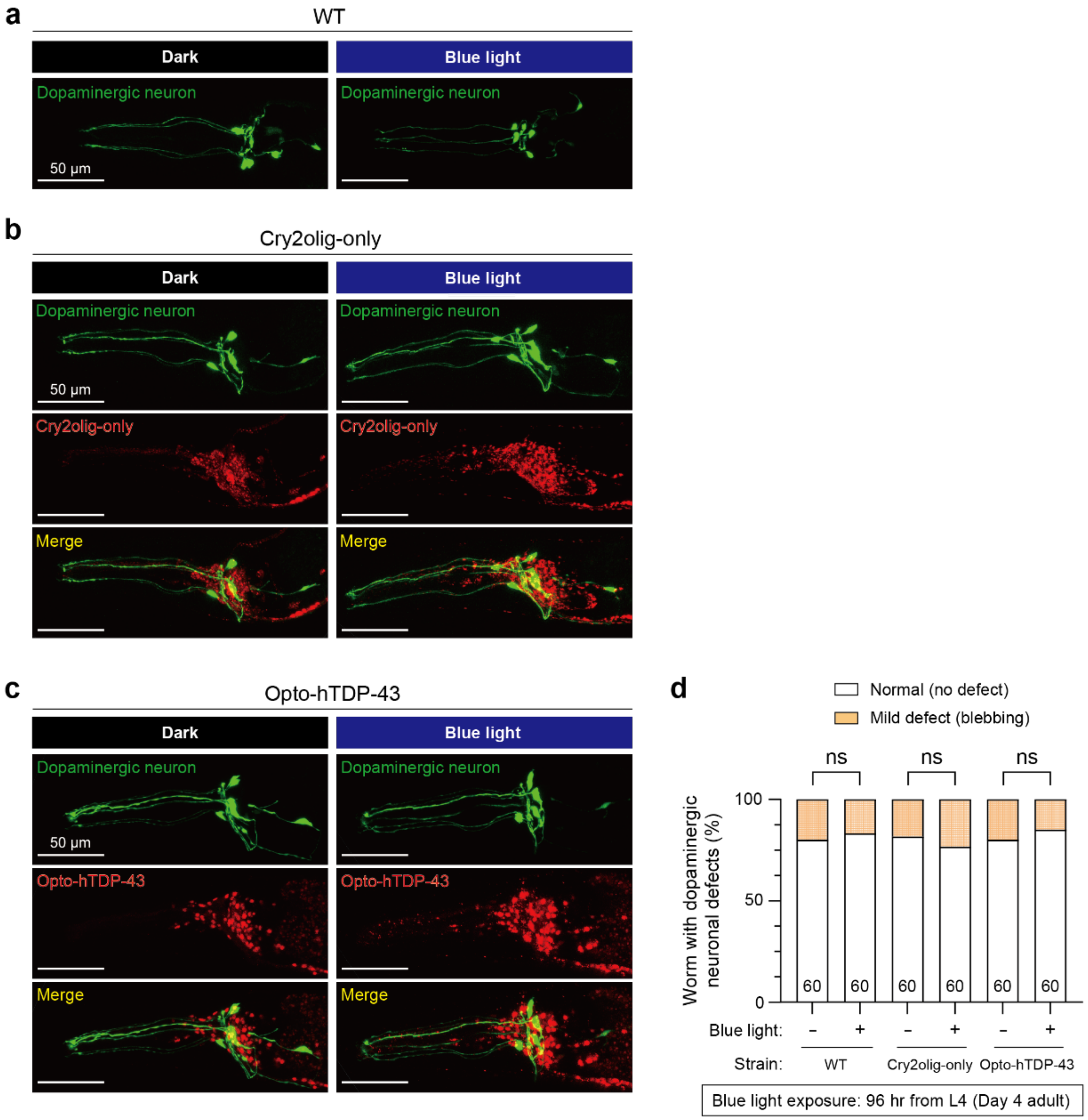

Figure S6. Cellular effects in dopaminergic neurons of opto-hTDP-43 expression under blue light stimulation.

(**a**) Confocal images of dopaminergic neurons in the head of WT worms before and after blue light stimulation. (**b**-**c**) Confocal images of dopaminergic neurons co-expressing mCherry: (**b**) Cry2olig-only and (**c**) Opto-hTDP-43. (**d**) Quantification of neuronal defects in dopaminergic neurons. Bar graph showing the percentage of worms with normal versus mild neuronal defects. Total sample size is indicated within each bar. Fisher's exact test. not significant (ns). All experiments were independently repeated twice.

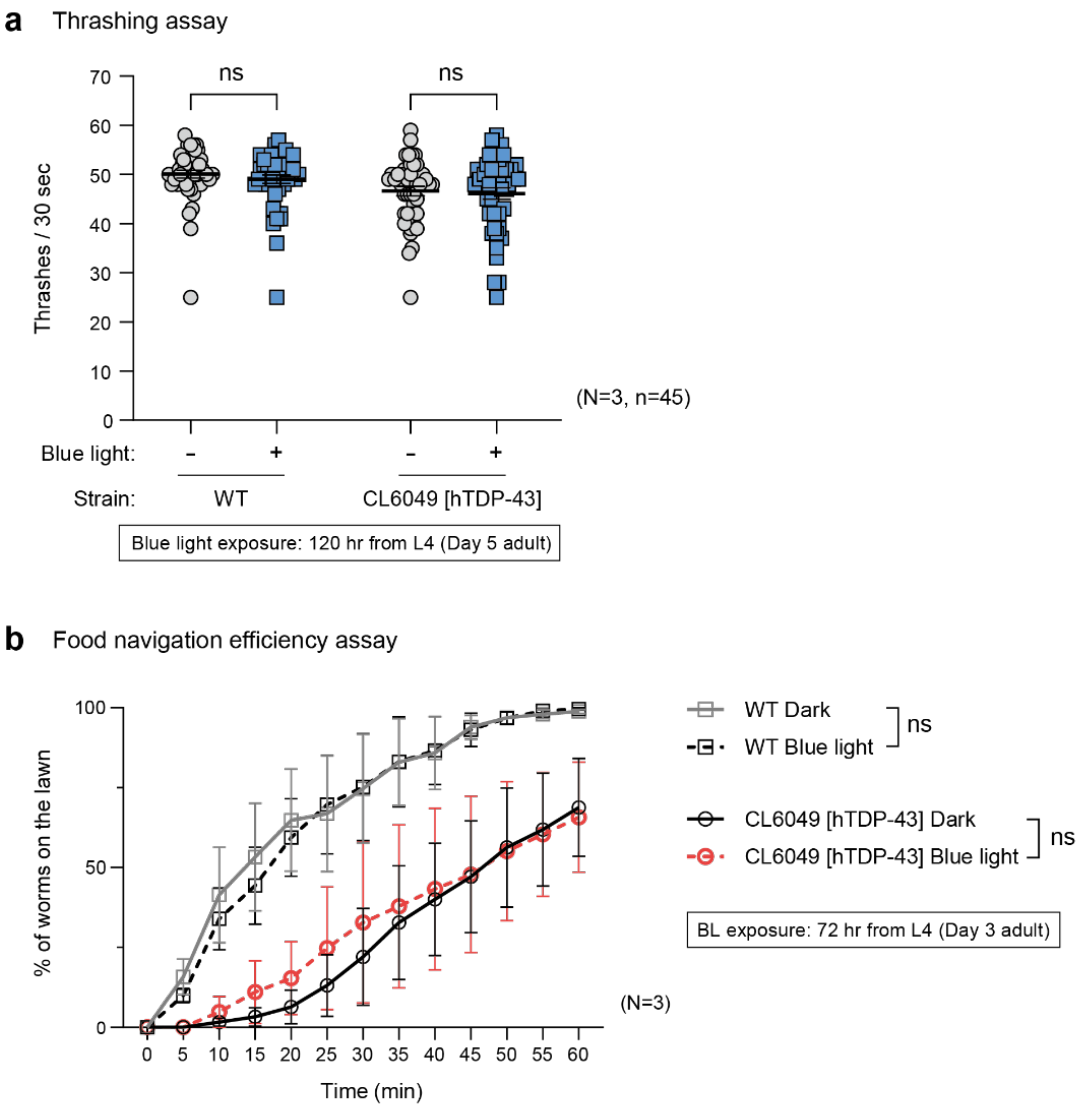

Figure S7. Assessment of phototoxicity on hTDP-43 protein
(**a**) Thrashing assay. Bar graph showing the average number of thrashes per 30 seconds for WT and hTDP-43-expressing worms under dark and blue light conditions. Data are presented as mean ± SEM. One-way ANOVA. not significant (ns). (**b**) Food navigation efficiency assay. Line graph depicting the percentage of worms on the bacterial lawn over time for each condition. More than 100 worms per strain per condition were analyzed in each experiment. Data are presented as mean ± SEM. Two-way ANOVA. ***$P < 0.001$, not significant (ns). All experiments were independently repeated three times.

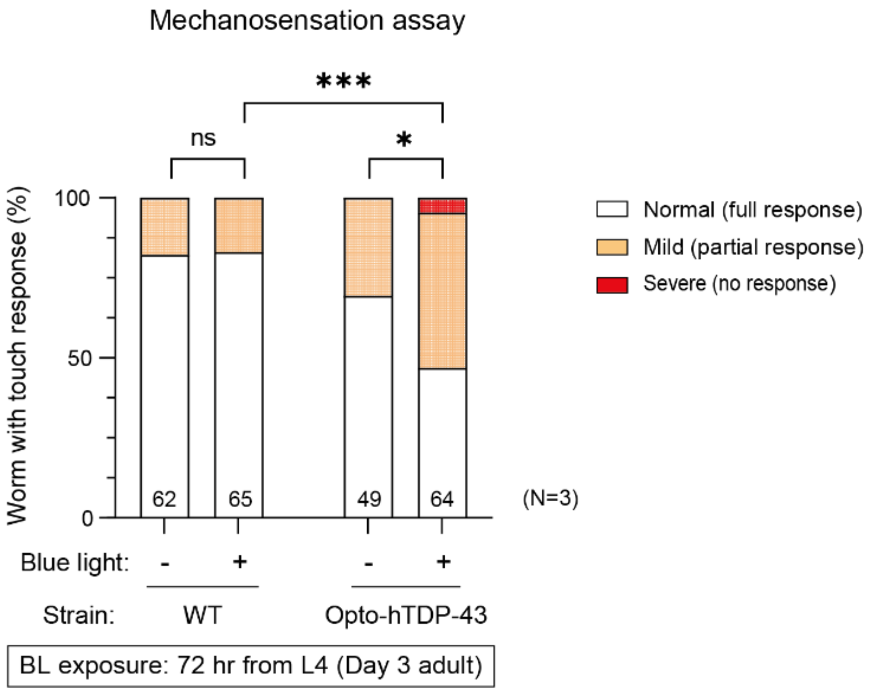

Figure S8. Mechanosensory defects in opto-hTDP-43-expressing worms under blue light stimulation.

Mechanosensation assay. Bar graph displaying the percentage of worms exhibiting normal, mild, and severe mechanosensory defects. Total sample size for each group is indicated within each bar. Fisher's exact test; $*P < 0.05$, $***P < 0.001$, not significant (ns). All experiments were independently repeated three times.

a **b**

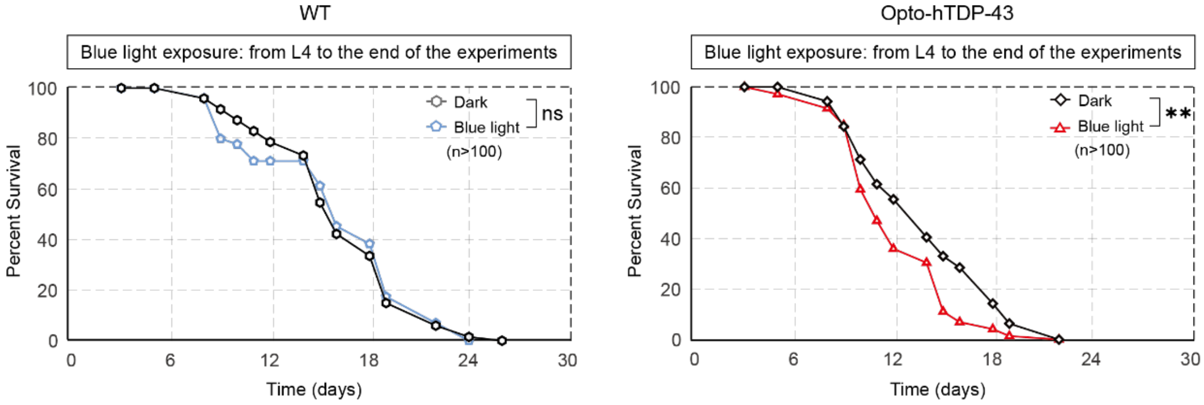

c

Average lifespan (days)

	WT	Opto-hTDP-43
Dark	16.21	13.86
Blue light	15.95	12.1

Figure S9. Lifespan-reducing effects of opto-hTDP-43 expression under blue light stimulation.

(**a**) Survival curves for WT worms under dark and blue light conditions. (**b**) Survival curves for opto-hTDP-43 worms. Log-rank test; $^{**}P < 0.01$, not significant (ns). (**c**) Table displaying a comparison of the average lifespan of WT and opto-hTDP-43 worms.

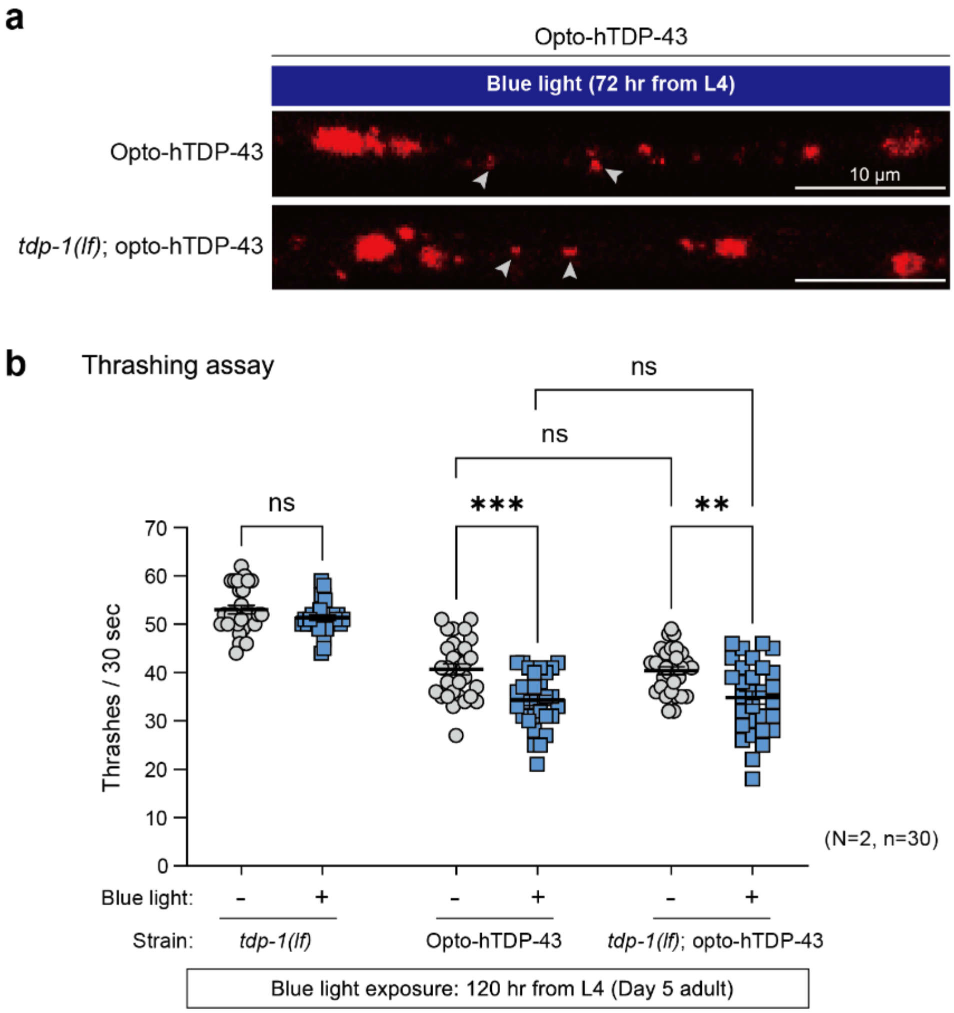

Figure S10. Lack of TDP-1 shows limited effect on motor function in opto-hTDP-43 worms. (**a**) Confocal imaging showing mCherry signal of opto-hTDP-43 condensates after illumination in opto-hTDP-43 and *tdp-1 (lf)*; opto-hTDP-43 worms. (**b**) Thrashing assay. Bar graph showing the average number of thrashes per 30 seconds for *tdp-1(ok803)*, opto-hTDP-43, and *tdp-1(ok803)*; opto-hTDP-43 transgenic worms under dark and blue light conditions. Data are presented as mean ± SEM. One-way ANOVA. $**P < 0.01$, $***P < 0.001$, not significant (ns). All experiments were independently repeated twice.

Table S1. ***C. elegans*** **strains used in this study**

C. elegans **strain**	**Source**	**Identifier**
Wild type N2	Caenorhabditis Genetics Center	Strain:N2
CZ13799 *juIs76[unc-25p::GFP] II*	Caenorhabditis Genetics Center	CZ13799
CZ631 *juIs14[acr-25p::GFP] IV*	Caenorhabditis Genetics Center	CZ631
CL6049 *dvIs62[snb-1p::hTDP-43] X*	Caenorhabditis Genetics Center	CL6049
RB929 *tdp-1(ok803)*	Caenorhabditis Genetics Center	RB929
BZ555 *egIs1[dat-1p::GFP]*	Caenorhabditis Genetics Center	BZ555
KWK75 *hluEx28[rgef-1p::mCherry::hTDP-43-Cry2olig]*	This paper	KWK75
KWK85 *hluEx35[rgef-1p::mCherry::Cry2olig]*	This paper	KWK85
KWK87 *hluEx38[rgef-1p::mCherry::hTDP-43]*	This paper	KWK87
KWK91 *hluIs5[rgef-1p::mCherry::hTDP-43::Cry2olig]*	This paper	KWK91
KWK92 *juIs76[unc-25p::GFP] II; hluIs5[rgef-1p::mCherry::hTDP-43::Cry2olig]*	This paper	KWK92
KWK123 *juIs14[acr-25p::GFP] IV; hluIs5[rgef-1p:: mCherry::hTDP-43::Cry2olig]*	This paper	KWK123
KWK124 *juIs76 [unc-25p::GFP] II; hluEx35[rgef-1p::mCherry::Cry2olig]*	This paper	KWK124
KWK125 *juIs14[acr-25p::GFP] IV; hluEx35[rgef-1p::mCherry::Cry2olig]*	This paper	KWK125
KWK132 *egIs1[dat-1p::GFP]; hluIs5[rgef-1p::mCherry::hTDP-43::Cry2olig]*	This paper	KWK132
KWK133 *egIs1[dat-1p::GFP]; hluEx35[rgef-1p::mCherry::Cry2olig]*	This paper	KWK133

Table S2. Plasmids used in this study

**Recombinant DNA**	**Identifier**
pCZGY1890: *unc-25p::gtwy::unc-54 3'UTR*	pCZGY1890
pKK119: *rgef-1p::FLAG::CEBP-1::GFP::unc-54 3'UTR*	pKK119
pCFJ90: *myo-2p::mCherry::unc-54 3'UTR*	pCFJ90
pRN24: *rgef-1p::mCherry::hTDP-43::Cry2olig*	pRN24
pRN29: *rgef-1p::mCherry::Cry2olig*	pRN29
pRN30: *rgef-1p::mCherry::hTDP-43*	pRN30

Table S3. Primers used in this study

Primer	Sequence
Primer for PCR: hTDP-43 Forward	5'-ATGTCTGAATATATTCGGGTAACCG-3'
Primer for PCR: hTDP-43 Reverse	5'-CTACATTCCCCAGCCAGAAG-3'
Primer for Gibson Cloning: *rgef-1* promoter Forward	5'-AGGAAACAGCTAAGCGGGCATAGATGATCCCATCG-3'
Primer for Gibson Cloning: *rgef-1* promoter Reverse	5'-ACCCTTTGAGACCATCGACGAACGATTGAGCAGAA-3'
Primer for Gibson Cloning: Cry2olig Forward	5'-CTGGGGAATGATGAAGATGGACAAAAAGACTATAG-3'
Primer for Gibson Cloning: Cry2olig Reverse	5'-GGTCGAATTCGCCCTTCTATGCTGCTCCGATCATGATCT-3'
Primer for Gibson Cloning: mCherry Forward	5'-CTCCGAATTCGCCCTATGGTCTCAAAGGGTGAAGA-3'
Primer for Gibson Cloning: mCherry Reverse	5'-AATATATTCAGACATCTTATACAATTCATCCATGCCACC-3'
Primer for qRT-PCR: *tdp-1* Forward	5'-GAAAGAATTGTCAGCAAAGGATCGC-3'
Primer for qRT-PCR: *tdp-1* Reverse	5'-GCCATAATCAGGGCCAACTGATTG-3'
Primer for qRT-PCR: human *TARDBP* Forward	5'-GGCAGCACTACAGAGCAGTTG-3'
Primer for qRT-PCR: human *TARDBP* Reverse	5'-AAGGCCTGGTTTGGCTCC-3'
Primer for qRT-PCR: *gpd-1/4* Forward	5'- GGACCAATGAAGGGAATCCTC -3'
Primer for qRT-PCR: *gpd-1/4* Reverse	5'- CATTGTCGTACCAAGAGACGAG -3'
